# Amphetamine Induces Sex-Dependent Loss of the Striatal Dopamine Transporter in Sensitized Mice

**DOI:** 10.1523/ENEURO.0491-23.2023

**Published:** 2024-01-10

**Authors:** Tarique Bagalkot, Alexander Sorkin

**Affiliations:** Department of Cell Biology, University of Pittsburgh School of Medicine, Pittsburgh 15261, Pennsylvania

**Keywords:** amphetamine, dopamine, endocytosis, trafficking, transporter

## Abstract

Dopamine transporter (DAT) controls dopamine signaling in the brain through the reuptake of synaptically released dopamine. DAT is a target of abused psychostimulants such as amphetamine (Amph). Acute Amph administration induces transient DAT endocytosis, which, among other Amph effects on dopaminergic neurons, elevates extracellular dopamine. However, the effects of repeated Amph abuse, leading to behavioral sensitization and drug addiction, on DAT are unknown. Hence, we developed a 14 d Amph-sensitization protocol in knock-in mice expressing HA-epitope-tagged DAT (HA-DAT) and investigated the effects of Amph challenge on sensitized HA-DAT animals. The Amph challenge resulted in the highest locomotor activity on Day 14 in both sexes, which was sustained for 1 h in male but not female mice. Strikingly, significant (by 30–60%) loss of the HA-DAT protein in the striatum was caused by the Amph challenge of sensitized males but not females. Amph also reduced *V*_max_ of dopamine transport in the striatal synaptosomes of males without changing *K*_m_ values. Consistently, immunofluorescence microscopy revealed a significant increase of HA-DAT colocalization with the endosomal protein VPS35 only in Amph-challenged males. Amph-induced loss of striatal HA-DAT in sensitized mice was blocked by chloroquine, vacuolin-1, and inhibitor of Rho-associated kinases ROCK1/2, indicative of the involvement of endocytic trafficking in the DAT protein loss. Interestingly, an apparent degradation of HA-DAT protein was observed in the nucleus accumbens and not in the dorsal striatum. We propose that Amph challenge in sensitized mice triggers Rho-mediated endocytosis and post-endocytic trafficking of DAT in a brain-region-specific and sex-dependent manner.

## Significance Statement

Dopamine transporter (DAT) is the major regulator of dopamine neurotransmission and is a direct target of abused psychostimulant amphetamine. However, how amphetamine affects DAT function remains poorly understood. Here, we studied the effects of repeated amphetamine administration, which leads to behavior and physiological sensitization, on DAT in mouse brain. We found that an acute amphetamine administration in sensitized mice causes a dramatic loss of DAT in the striatal axons of male but not female mice, and that this DAT protein loss is due to DAT endocytosis. To our knowledge, this is the first demonstration of amphetamine-induced endocytic downregulation of DAT and its sex-dependence in an in vivo experimental model that recapitulates the development of human addiction behavior.

## Introduction

Dopamine (DA) signaling in the central nervous system plays a key role in modulating cognition, locomotion, motivation, and reward-seeking behaviors (Giros and Caron, 1993; Dani and Zhou, 2004). Dysregulation of DA neurotransmission is linked to neurodegenerative and neuropsychiatric disorders, including Parkinson's disease, schizophrenia, attention-deficit/hyperactivity disorder, autism spectrum disorder, and substance use disorder (Snyder, 2002; Gainetdinov and Caron, 2003; Iversen and Iversen, 2007; Wise, 2008; Gowrishankar et al., 2014; Volkow and Morales, 2015). DA neurotransmission is tightly controlled by the reuptake of synaptically released DA through the plasma membrane DA transporter (DAT). DAT is exclusively expressed in dopaminergic neurons. The proper level of DAT in the plasma membrane of dopaminergic axons is essential for the efficient removal of extracellular DA and to control the amplitude and duration of DA neurotransmission. DAT is a major target of abused psychostimulants, such as amphetamines (Amph) and cocaine, which are substrates and a competitive antagonist of DAT, respectively. However, the mechanisms by which these compounds affect DAT function and its subcellular distribution are not fully understood.

The abuse potential and psychomotor stimulant properties of Amph have been linked to its ability to elevate DA concentrations in the striatum. The resulting increased dopaminergic input in the striatum, mainly in the ventral striatum including nucleus accumbens (NAc), has been associated with the rewarding properties of Amph (Segal and Kuczenski, 1992; Paulson and Robinson, 1995; Pierce and Kalivas, 1995; Schultz, 2002; Sulzer et al., 2005). Several mechanisms, such as increased DA release, competitive inhibition of DA reuptake, and conversion of DAT to a substrate efflux mode, have been implicated in Amph-induced increase in extracellular DA concentration (Khoshbouei et al., 2003; Sulzer et al., 2005; Robertson et al., 2009). Acute (one-time) treatment with Amph has also been shown to reduce DAT surface levels by inducing DAT endocytosis in acute midbrain (MB) slices as measured using slice biotinylation (Wheeler et al., 2015), cultured dopaminergic neurons (Hong and Amara, 2013), striatal synaptosomes (Johnson et al., 2005; Richards and Zahniser, 2009), and heterologous cells (Saunders et al., 2000; Chi and Reith, 2003; Sorkina et al., 2003; Kahlig et al., 2004). Various mechanisms have been implicated in Amph-induced DAT endocytosis, including the activation of protein kinase C (PKC) (Chen et al., 2009), Akt (Garcia et al., 2005), and RhoA GTPase (Wheeler et al., 2015). By contrast, our studies using fluorescence and electron microscopy (Block et al., 2015) and others (German et al., 2012) observed that acute Amph treatment does not stimulate DAT endocytosis in the dorsal striatum (dStr) and midbrain in the intact mouse brain and acute brain slices ex vivo.

However, relatively little is known regarding DAT endocytosis and trafficking in the intact brain during repetitive Amph exposure. Repetitive abuse of Amph results in a progressive amplification in the behavioral and neurochemical response, a phenomenon termed sensitization (Robinson and Becker, 1986). Sensitization may persist months after administration, thus mimicking long-term sensitivity to drugs as observed in human addicts. At the neurochemical level, sensitization to Amph is manifested in lasting hyper-responsiveness of dopaminergic pathways, which correlates with an increase in the concentration of extracellular DA after a given dose of Amph in rodents (Kalivas and Duffy, 1993; Wolf et al., 1993; Paulson and Robinson, 1995; Pierce and Kalivas, 1995; Robinson et al., 1998; Robinson and Badiani, 1998) and humans (Boileau et al., 2006; Booij et al., 2016). In the present study, we explored the effects of Amph challenge on DAT in Amph-sensitized dopaminergic neurons. To this end, we developed an Amph-sensitization protocol in knock-in mice expressing HA-epitope-tagged DAT (HA-DAT) (Rao et al., 2012). The analysis using multiple complementary approaches demonstrated a rapid Amph-induced decrease in the amount of HA-DAT in the striatum of Amph-sensitized mice, which involves endocytic trafficking and is dependent on RhoA effector, Rho-associated kinases (ROCK1/2) activity. Surprisingly, this apparent degradation of HA-DAT was strictly sex-dependent as it was observed in male but not female animals.

## Materials and Methods

### Animals

Male and female (7–8 weeks of age; weight, 20–22 g) HA-DAT knock-in mice (on the C57BL/6J background; Rao et al., 2012, 2013) were used in this study. During experiments, all mice were single-housed in cages in a regulated environment (23 ± 1°C, 50 ± 5% humidity) on a 12 h light/12 h dark cycle and were fed ad libitum. All procedures were conducted in accordance with the National Institutes of Health's *Guide for the care and use of laboratory animals* and with the approval of the Institutional Animal Care and Use Committee of the University of Pittsburgh.

### Antibodies and chemicals

Antibodies were purchased from the following sources: the mouse monoclonal antibody against the HA11 epitope (16B12) were from BioLegend (mms-101p), and the rat monoclonal antibody against the N-terminus of DAT (MAB369) and rabbit polyclonal antibody against tyrosine hydroxylase (TH) (AB152) were from EMD Millipore. The rabbit polyclonal antibodies against vacuolar protein sorting-associated protein 35 (VPS35) were from Novus Biologicals (NB100-1397). The rabbit polyclonal antibodies against vacuolar protein sorting-associated protein 26 (VPS26) was kindly provided by Dr. J. Bonifacino (National Institute of Child Health and Disease). The mouse monoclonal antibody to vesicular monoamine transporter 2 (VMAT2) were from Santa Cruz Biotechnology (sc-374079). The rabbit polyclonal antibodies against autophagy-related protein 9A (ATG9) were from Novus Biologicals (NB110-56893SS). Secondary IRDye-800 and IRDye-680-conjugated goat anti-mouse IgG1, anti-rat, and anti-rabbit antibodies were purchased from LI-COR Biosciences; AlexaFluor-488 (A488)- and Cy5-conjugated donkey anti-mouse and anti-rabbit secondary antibodies were from Jackson ImmunoResearch Laboratories. Paraformaldehyde (PFA) was from Electron Microscopy Sciences. All other reagents and supplies were from Thermo Fisher Scientific or Sigmachem unless noted otherwise.

### Amph-sensitization protocol

**Figure 1.**
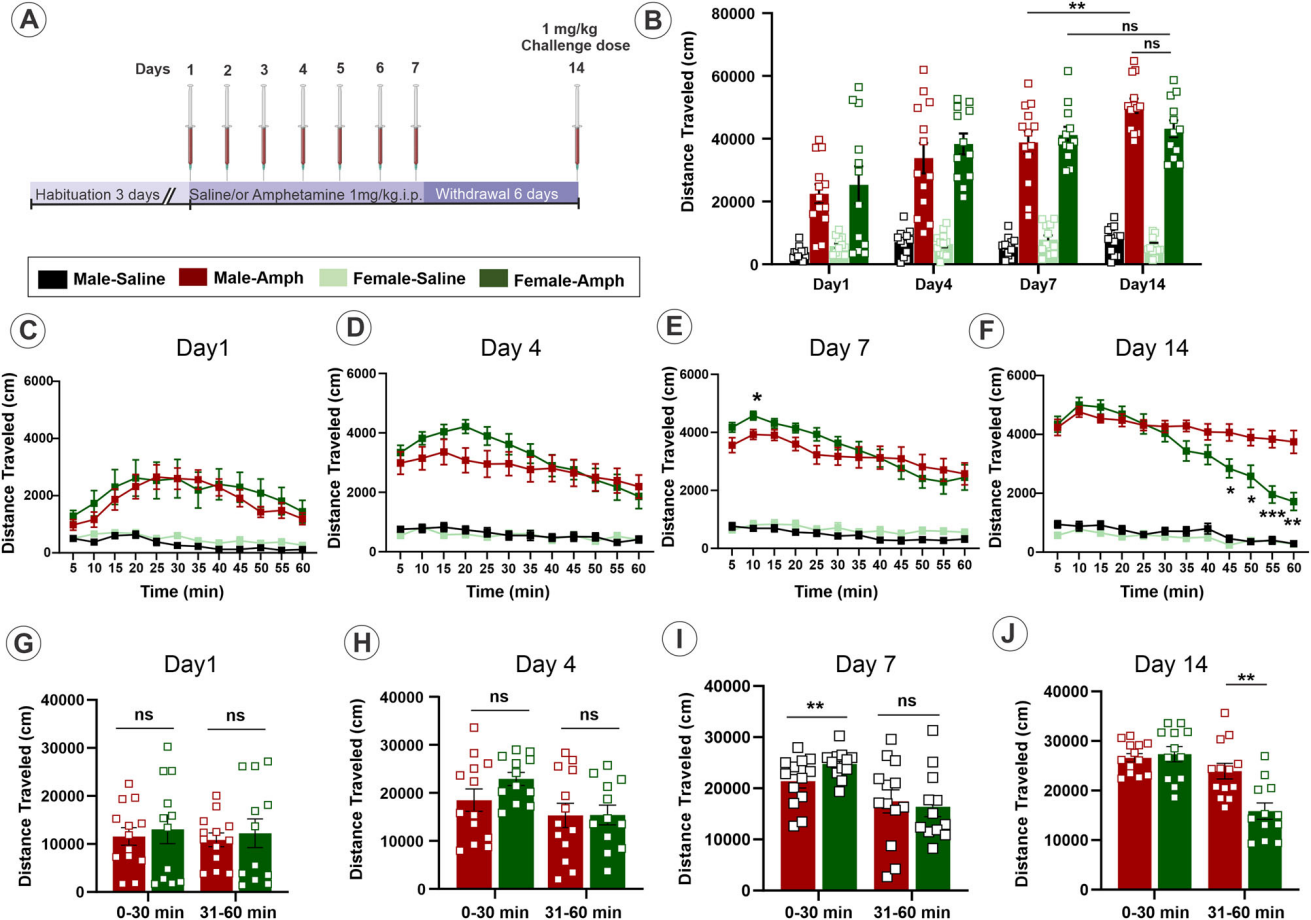
Repeated administration of Amph results in sustained behavioral sensitization in male mice. ***A***, Schematics of the Amph-sensitization protocol. On all days, animals were injected i.p. after a 60 min habituation in locomotion chambers. ***B***, Locomotion activity was measured for 1 h after saline or Amph injection on Day 1, 4, 7, and after Amph challenge on Day 14. A two-way repeated measure ANOVA analysis showed a significant main effect of group (*F*_(3,46) _= 70.44, *p* < 0.0001), day (*F*_(2.232, 102.7)_ = 27.90, *p *< 0.0001), and interaction of group × day (*F*_(9,138)_ = 7.729, *p *= 0.000). Tukey's post hoc analysis revealed highest locomotor activity on Day 7 and 14 of AMPH-treated male and female mice compared with respective saline groups (Male: Day 7, *p* = 0.003 and Day 14, *p *< 0.0001, and Female: *p *< 0.0001 and *p *< 0.0001, respectively), and robust behavioral sensitization was observed in sensitized male mice on Day 14 compared with Day 7 (*p* > 0.0047), but not in female (*p* = 0.8076). ***C***, Time-course of the locomotion activity during a 1 h Amph challenge on Day 1. A two-way repeated measure ANOVA analysis showed a significant main effect of group (*F*_(3,46) _= 11.42, *p *< 0.000), time (*F*_(3.333,153.3)_ = 13.69, *p *< 0.000), and interaction of group × time (*F*_(33,506) _= 4.503, *p* < 0.000). Tukey's post hoc analysis revealed an increase in locomotor activity in Amph-challenged male and female mice compared with their respective controls. ***D***, Time-course of the locomotion activity during a 1 h Amph challenge on Day 4. A two-way repeated measure ANOVA analysis showed a significant main effect of group (*F*_(3,46) _= 30.29, *p *< 0.000), time (*F*_(4.742, 218.1)_ = 27.34, *p *< 0.000), and interaction of group × time (*F*_(33,506) _= 6.438, *p *< 0.000). Tukey's post hoc analysis revealed an increase in the locomotor activity in Amph-challenged male and female mice compared with their respective controls. ***E***, Time-course of the locomotion activity during a 1 h Amph challenge on Day 7. A two-way repeated measure ANOVA analysis showed a significant main effect of group (*F*_(3, 46)_ = 66.35, *p *< 0.000), time (*F*_(3.740, 172.1)_ = 30.87, *p *< 0.000), and interaction of group × time (*F*_(33,506)_ = 6.210, *p *< 0.000). Tukey's post hoc analysis revealed an increase in the locomotor activity in Amph-challenged male and female mice compared with their respective controls. ***F***, Time-course of the locomotion activity during a 1 h Amph challenge on Day 14. A two-way repeated measure ANOVA analysis showed a significant main effect of group (*F*_(3,46) _= 150.4, *p *< 0.0001), day (*F*_(3.850, 177.1)_ = 35.94, *p *< 0.0001), and interaction of group × day (*F*_(33,506)_ = 10.13, *p *= 0.000). Tukey's post hoc analysis revealed a decrease in the locomotor activity in the AMPH-challenged female mice compared with males at 45, 50, 55, and 60 min. *p < 0.05, **p < 0.01, and ***p < 0.001 indicate significant difference between Amph-sensitized male and female mice. ***G*–*J***, 1 h locomotion test: the total distance traveled by Amph-sensitized male and female mice for the first 30 min and the second 30 min at corresponding days of the sensitization protocol. Error bars are SEMs. *p < 0.05 and **p < 0.01 indicate significant difference between Amph-sensitized male and female mice. ns, no significant difference. See Extended data Figure 1-1 for further details on the effects of Amph on the total time spent engaged in stereotypical behavior on Days 1, 4, 7, and 14.

The D-Amphetamine hemisulfate (Amph) (Sigma) was dissolved in normal saline and administered intraperitoneally (i.p.). Amph (0.1 mg/ml) was administered at a dose of 1 mg/kg for sensitization by daily injections for 7 consecutive days (Fig. 1*A*). Equivalent volumes of saline were injected in control mice. After the 7 d withdrawal, mice were challenged on Day 14 with saline or Amph (1 mg/kg). A 1 mg/kg of Amph was chosen because a low dose of Amph enhances locomotor activity (Shen et al., 2001; Gatica et al., 2020) whereas a high dose leads to stereotypical behaviors (Shen et al., 2001; Chinen et al., 2006). For behavior experiments, locomotor activity was recorded on Days 1, 4, 7, and 14. For biochemical experiments, mice were euthanized with isoflurane inhalation followed by decapitation and brain removal on Day 7, after withdrawal (7DW) on Day 14 before and after 1 h challenge injections with saline or Amph. Different animals were used for behavior and biochemical experiments. To separate the effects of novelty from the pharmacological effects of the drug in behavior experiments, the animals were acclimated for 3 d (habituation) to the testing room and locomotor chambers and injected with saline prior to the sensitization protocol. For biochemical experiments, the mice were not habituated to the behavior testing room or locomotor chambers but received a saline injection for 3 d of habituation period (Fig. 1*A*).

10.1523/ENEURO.0491-23.2023.f1-1 Figure 1-1 **Repeated administration of Amph results in decreased in time spent showing stereotypy-like behavior in sensitized mice.** Stereotypy time was measured for 1 hr after saline or Amph injection on day 1, 4, 7 and after Amph challenge on day 14. Two-way repeated measures ANOVA analysis showed a significant main effect of group (*F*_3,46_ = 58.06, *p*˂0.0001), day (*F*_2.911, 133.9_= 5.398, *p*˂0.001), and interaction of group x day (*F*_9,138_= 4.571, *p*˂0.0001). *Tukey’s post hoc* analysis revealed decreased stereotypy time spent on day 1, 4, 7 and 14 of AMPH treated male and female mice compared to respective saline groups (Male: Day 1; p=0.008, Day 4; p˂0.000, Day 7; p˂0.001 and Day 14; p˂0.0001, and Female: p=0.008, p˂0.0001, p˂0.0001 and p˂0.0001, respectively). Download Figure 1-1, TIF file.

### Behavioral tests

The behavioral tests were conducted in the Rodent Behavior Analysis Core of the University of Pittsburgh Schools of Health Sciences. The locomotor activity (distance traveled) and time spent showing stereotypy-like behavior (stereotypy time) was quantified using an open-field arena (40 × 40 × 40 cm; Omnitech Electronics) in a sound-attenuated and environmentally controlled chamber (60 × 64 × 60 cm; Omnitech Electronics) equipped with 4 horizontal 16 × 16 arrays of infrared photobeam sensors. Measurements were conducted in 5 min bins for 60 min after Amph or saline injection on experimental Days 1, 4, 7, and 14, by using behavioral tracking software (Fusion, Omnitech Electronics). On each test day, the animals were acclimated to individual activity chambers for 60 min to allow the animal to become accustomed to its behavioral cage before subsequent injections of either Amph (1 mg/kg, i.p.) or saline. Following each injection, the mice were placed back into their respective activity chambers, and their locomotor activity was recorded.

### Striatal synaptosome preparation

The synaptosomes were prepared as described (Sorkina et al., 2018). Briefly, striatal tissue was rinsed in ice-cold Gey's balanced salt solution with 10 mM D-glucose. The striatum was homogenized in 1.5 ml of ice-cold HEPES buffer (5 mM HEPES, 0.32 M sucrose, pH 7.4) using a glass homogenizer. The homogenate was centrifuged at 1,000 × *g* for 10 min at 4°C, and the resulting supernatant was centrifuged at 12,500 × *g* for 20 min at 4°C to pellet the synaptosomes. Pellets were resuspended in ice-cold Krebs-Ringer HEPES buffer (KRH; 120 mM NaCl, 4.7 mM KCl, 2.2 mM CaCl_2_, 1.2 mM MgSO_4_, 1.2 mM KH_2_PO_4_, 10 mM glucose, 10 mM HEPES, pH 7.4) and used for the Western blot analysis or DA uptake measurements.

### Synaptosome DA uptake

The synaptosomes from two mice for each individual experiment and pellets were resuspended in KRH. The synaptosomes were incubated with 20 nM [^3^H]DA (Perkin Elmer Life Sciences) and unlabeled DA (0.031, 0.0625, 0.125, 0.25, 0.5, and 1.0 µM) for 10 min at 37°C in KRH supplemented with 10 µM ascorbic acid, 10 µM pargyline, 1 µm desipramine, and 10 µm cathechol-O-methyl-transferase inhibitor. Nonspecific [^3^H]DA accumulation was determined in the presence of 100 µM cocaine. The reaction was terminated by adding ice-cold KRH and immediate washing with KRH by filtration through Whatman GF/A glass fiber filters using a Brandel cell harvester (Model M-48 Biochemical Research and Development Laboratories). The filters were then solubilized in 0.1N NaOH/1% SDS. Synaptosomes-associated [^3^H]DA was measured by liquid scintillation counting. The kinetic parameters (*K*_m_ and *V*_max_) for [^3^H]DA uptake were calculated using the Michaelis–Menten equation by GraphPad software.

### Western blotting

The mice were euthanized, and the brains were rapidly extracted and sliced into coronal sections (0.4 mm thick) using a brain matrix followed by either preparation of synaptosomes from the striatum as described above or extraction of tissue from specific brain regions. Tissues from dStr, NAc, ventral tegmental area (VTA), and substantia nigra compacta (SNc) were extracted using a 1.0 mm Harris Uni-Core micropunch (Electron Microscopy Sciences). Synaptosomes and brain tissues were solubilized in the lysis buffer containing 50 mM Tris-HCl pH 7.4, 0.5% sodium deoxycholate, 1% NP-40, 150 mM NaCl, 2 mM EGTA, 2 mM EDTA, and phosphatase and protease inhibitors. Lysates were cleared by centrifugation for 15 min at 16,000 × *g* at 4°C. Aliquots of lysates (30 µg of protein for striatal synaptosomes, dStr, and NAc; 50 µg for whole MB, VTA, and SNc) were denatured in sample buffer at 37°C for 30 min. Lysates were resolved by 7.5% SDS-PAGE, transferred to nitrocellulose (Li-COR), and probed with appropriate primary and secondary antibodies conjugated to far-red fluorescent dyes (IRDye-680 or IRDye-800) followed by detection using Odyssey Li-COR system. Quantifications were performed using ImageJ software.

### Immunofluorescence staining

Mice were injected intraperitoneally with xylazine/ketamine, and perfused transcardially with 50 ml of PBS and 50 ml of 4% PFA in PBS (pH 7.2). The brains were then post-fixed with 4% PFA for 4 h at 4°C and cryoprotected in 20% sucrose/TBS overnight followed by 30% sucrose in PBS at 4°C until ready for cryosectioning. The brains were embedded in OCT compound (Sakura Finetek), and deep frozen in liquid nitrogen. Sagittal free-floating brain sections were made at 50 µm thickness using freezing microtome (Leica CM 1950). The sections were incubated in PBS containing 1% H_2_O_2_ for 15 min, permeabilized with 0.1% Triton X-100 for 1 h, and preincubated with blocking buffer containing 10% normal donkey serum (D9663, Sigma Millipore), 3% BSA (A2153, Sigma Millipore), and 0.1% Triton X-100 (Sigma Millipore) in PBS for 1 h at room temperature. After blocking, the sections were incubated with mouse HA11 (1:500) and rabbit VPS35 (1:500) or rabbit ATG9 antibodies at 4°C in a blocking solution for 48 h and washed, followed by donkey anti-mouse conjugated to Alexa488 and Cy5-conjugated donkey anti-rabbit secondary antibodies for 1 h at room temperature. Nuclei were stained with Hoechst 33342 (62249, Thermo Fisher Scientific). The sections were mounted in a ProLong Gold antifade mounting medium (P36930, Thermo Fisher Scientific).

### Confocal microscopy and image analysis

High-resolution z-stacks of confocal images of DA neurons in sagittal sections were acquired using a spinning disk confocal Marianas system (Intelligent Imaging Innovation) as described (Bagalkot et al., 2021). Typically, 20–30 serial two-dimensional confocal images of cryosections were recorded at 400 nm intervals. All image acquisition settings were identical for all experimental variants in each experiment.

To quantify the amount of HA-DAT colocalized with VPS35, 3D images were cropped to generate new images consisting of a z-stack of three consecutive confocal sections that displayed similar fluorescence intensity (similar antibody penetration) among variants in each individual experiment and that were away from the surface of a section where significant nonspecific fluorescence was typically present. Cropped 3D images deconvolved using a No Neighbors algorithm of SlideBook 6. An automated segment mask was generated from background-subtracted images to select voxels detected through the 488 nm channel (Mask 1; HA-DAT) and the 640 channels (Mask 2; VPS35). A “colocalization” mask (Mask 3) was generated to select voxels positive for both 488 nm and 640 nm channels. The sum fluorescence intensity of the 488 nm channel in the colocalization mask was divided by the sum fluorescence intensity of HA-DAT (Mask 1) to calculate the fraction of total HA-DAT immunofluorescence colocalized with VPS35 per field of view.

### Experimental design and statistical analysis

The number of samples used in the experiments was determined to achieve power sufficient for establishing the statistical significance between tested variants. All statistical analyses were performed using GraphPad Prism software (GraphPad). For comparisons of each two groups, unpaired Student's *t* test was employed, and for comparisons of more than two groups, one-way ANOVA followed by Tukey's multiple comparison test was used. For behavior study, a two-way repeated measure ANOVA followed by Tukey's or Holm–Sidak's multiple comparison test was used. All experiments were performed at least three times. Differences were considered significant when the *p*-value was < 0.05, with the specific *p*-values detailed within each figure legend.

## Results

### Amph-induced locomotor sensitization

To test whether the repeated administration of Amph results in sensitization, we developed a schedule of Amph administration that includes repeated injections and withdrawal. HA-DAT mice were injected with saline or Amph (1 mg/kg, i.p.) for 7 d followed by 6 d of withdrawal and then challenged with saline or a single Amph dose (1.0 mg/kg, i.p.) on Day 14 (Fig. 1*A*). Because female and male rodents were previously shown to respond differently to Amph (Becker et al., 2001; Dluzen and McDermott, 2008; Bourque et al., 2011), we performed these experiments in mice of both sexes. Development of the behavior sensitization was evident by the highest locomotor activity measured during 1 h Amph challenge on Day 14 in both sexes (Fig. 1*B*). The two-way ANOVA for repeated measures indicated a significant effect of group (*F*_(3.46)_ = 70.44, *p *< 0.0001), day (*F*_(2.232, 102.7)_ = 27.90, *p *< 0.0001), and interaction of group × day (*F*_(9,138) _= 7.729, *p *= 0.0001)]. Tukey's test revealed that the Amph-treated male and female mice were significantly different from the saline-treated mice. However, the response was significantly stronger on Day 14 compared with Day 7 in the Amph-treated male but not female mice (Fig. 1*B*). Interestingly, on Day 1, it took 25 min for the locomotion to reach maximal stimulation, whereas on later days, maximum activity was observed after 5 min following Amph administration, indicative of a gradual sensitization (Fig. 1*C–F*). On Day 14, both sexes manifested high initial activity upon the Amph challenge, followed by a statistically significant reduction in activity after 30 min in female mice but not in male mice (Fig. 1*F*). Such sex-specific differences in sustaining high locomotion activity were not observed after single doses of Amph at earlier time points of the sensitization schedule, although acute Amph stimulation of the behavior was evident (Fig. 1*C,D,F–J*). Furthermore, the analysis of the total time spent engaged in stereotypical behavior showed that Amph-sensitized mice of both sexes exhibited a significant decrease in this parameter (Extended data Fig. 1-1). Thus, this data indicates that the reduction in locomotor activity in female mice after 30 min is not due to a shift from hyperlocomotion to stereotypy in response to Amph.

### Amph challenge of Amph-sensitized mice triggers loss of the HA-DAT protein in the striatum in a sex-dependent manner

**Figure 2.**
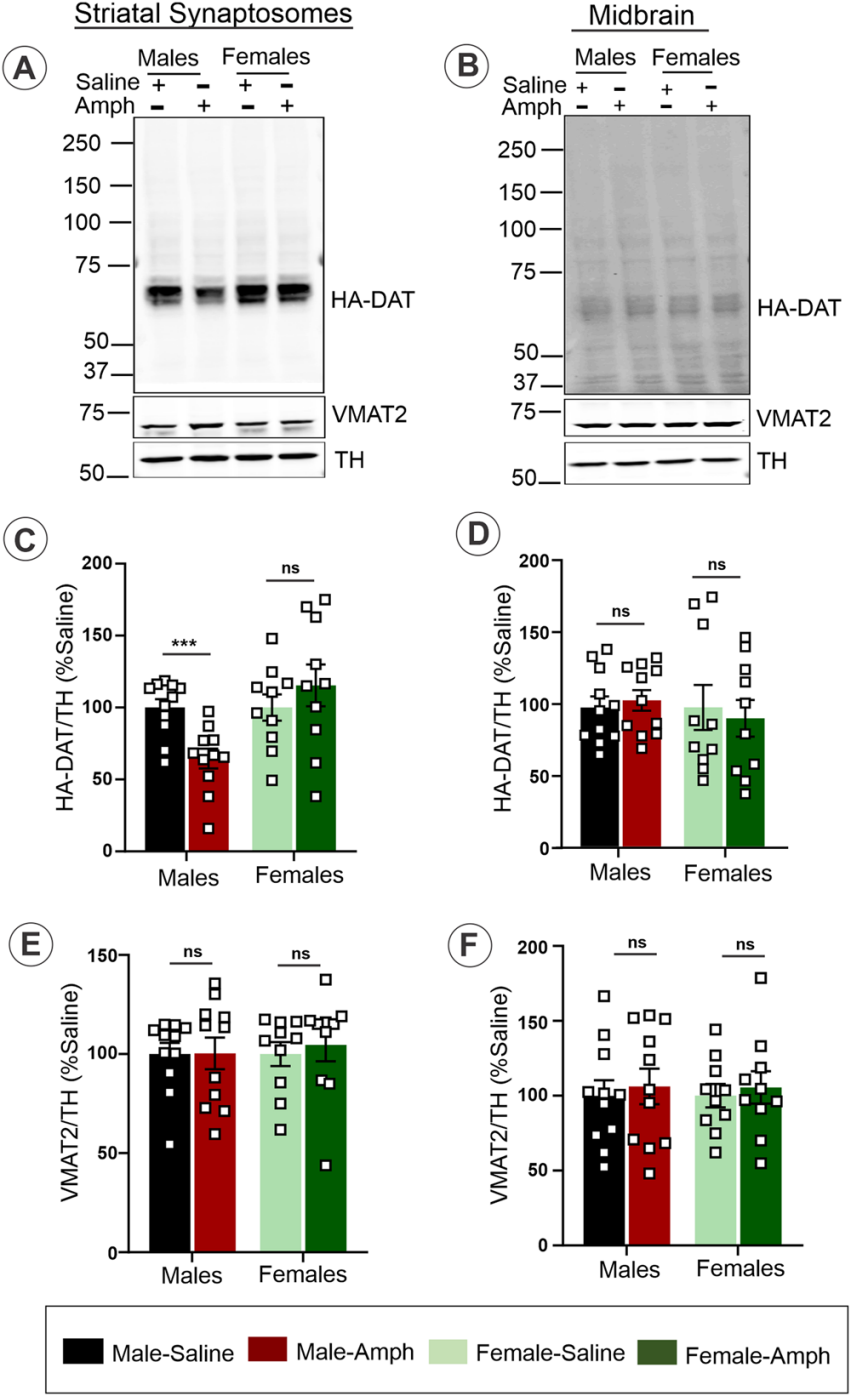
Amph challenge of Amph-sensitized mice decreases the DAT protein level in the striatum in sex-dependent manner. HA-DAT mice sensitized according to the protocol in Figure 1*A* were challenged with saline or Amph for 1 h on Day 14. Striatal synaptosomes (30 µg/lane) (***A*–*C***) and total midbrain tissue (50 µg/lane) (***D*–*F***) were lysed, and the aliquots of lysates (***C*,*F***) were resolved by 7.5% SDS-PAGE, transferred to nitrocellulose and probed with rat DAT, rabbit TH, and rabbit VMAT2 antibodies by Western blotting. Representative blots are shown. Bar graphs show mean values (±S.E.M) of the amounts of HA-DAT (***B*,*E***) or VMAT2 (***C,F***) normalized by the amount of TH and expressed per brain as a percentage of the normalized amount in saline samples. The data are from three or more independent experiments. Asterisks indicate significant differences of “Amph” samples compared with Saline groups, ***p < 0.001 [Student's unpaired *t* test (*n* = 10–11)]. ns, no significant difference. See Extended data Figure 2-1 for confirmation of the HA-DAT loss in male striatum using immunoblotting with the HA antibody.

To investigate the mechanisms underlying behavior sensitization described in Figure 1, we examined whether Amph sensitization affects DAT, a transporter that Amph uses to enter dopaminergic neurons. Hence, HA-DAT protein levels were measured by Western blotting with the antibody recognizing the amino-terminal region of DAT in the striatal synaptosomal preparations and MB homogenates obtained on Day 14 from saline- or Amph-challenged male and female mice. The level of tyrosine hydroxylase (TH) did not change during Amph sensitization and challenge, indicating that Amph administration did not have a general toxicity effect on dopaminergic neurons. Therefore, TH immunoreactivity was used as a normalization factor for DAT immunoreactivity. HA-DAT protein level in the striatal synaptosomes in Amph-sensitized male mice was found to be significantly reduced compared with saline-treated mice (*t* test: *t* = 3.9414, df = 20, *p* = 0.0008) (Fig. 2*A,B*). To rule out the possibility of Amph-induced conformational changes or post-translational modifications interfering with the recognition of the amino-terminal domain of DAT by the antibody, the decrease in the HA-DAT protein in males was demonstrated using the HA antibody (Extended data Fig. 2-1). By contrast, no difference in the HA-DAT amount was detected between saline and Amph-challenged female mice (*t* test: *t* = 0.8994, df = 18, *p* = 0.3803) (Fig. 2*A,B*). The amount of HA-DAT in MB lysates was not affected by the Amph challenge in sensitized male (*t* test: *t* = 0.4704, df = 20, *p* = 0.6431) and female mice (*t* test: *t* = 0.3718, df = 18, *p* = 0.7144) (Fig. 2*D,E*). The amount of vesicular monoamine transporter 2 (VMAT2), a transporter that is responsible for the accumulation of Amph in the synaptic vesicles of dopaminergic neurons, was not affected in the striatal synaptosomes (Fig. 2*A,C*) and MB lysates by the Amph challenge (Fig. 2*D,F*). These data demonstrate a high level of the specificity of Amph effects for DAT in sensitized male mice.

10.1523/ENEURO.0491-23.2023.f2-1 Figure 2-1 **Amph challenge of Amph-sensitized mice decreases HA-DAT protein level in striatum of males.** Striatal synaptosomes obtained after saline and Amph-challenged on Day 14 from male mice were lysed as in Figure 2A. The protein samples [60 µg/ lane of striatal synaptosomes] were resolved by 7.5% SDS-PAGE, transferred to nitrocellulose and probed with HA and TH antibodies by Western blotting. Representative blot is shown. The bar graph shows the mean (± S.E.M) intensity of bands from 3-4 independent experiments. The amounts of HA-DAT were normalized to the amounts of TH and expressed as percent of the normalized amount in Saline samples. Asterisks indicate significant differences compared to Saline group, *p ˂ 0.05 (Student’s unpaired t-test [n = 5]). Download Figure 2-1, TIF file.

**Figure 3.**
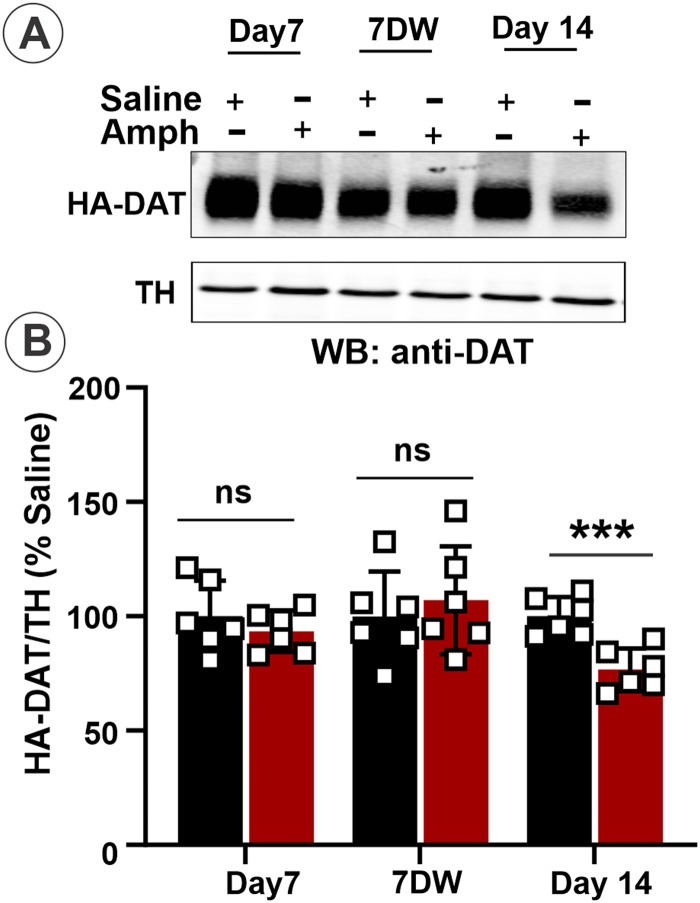
Amph challenge results in the partial loss of the HA-DAT protein in the striatal synaptosomes of male mice only on Day 14 of the sensitization protocol. ***A***, Striatal synaptosomes obtained on Day 7 and Day 14 before saline and Amph challenge (*7DW*), and after saline and Amph challenge on Day 14 were lysed. The protein samples (30 and 60 µg/lane) for DAT from striatal synaptosomes were resolved by 7.5% SDS-PAGE, transferred to nitrocellulose, and probed with DAT and TH antibodies by Western blotting. Representative blot is shown. ***B***, The bar graph shows the mean with SEM. Intensity of bands quantitated from three to four independent experiments, and the amounts of HA-DAT were normalized by TH and expressed as a percentage of the normalized amount in Saline samples. Asterisks indicate significant differences compared with the Saline group, ***p < 0.001 [Student's unpaired *t* test (*n* = 6)]. ns indicates no significant difference.

To confirm that a partial loss of HA-DAT in the striatal synaptosomes on Day 14 caused by the Amph challenge was specific for 14 d sensitized male mice, we measured HA-DAT expression levels in Amph-injected mice on Day 7 and Day 14 after withdrawal but before the 1 h Amph challenge (Fig. 3). The effects of Amph on Day 1 were not analyzed because we have not observed any significant effects of a single Amph injection on HA-DAT localization in the striatum and MB in previous studies (Block et al., 2015). The Western blot analysis showed no difference in the amount of HA-DAT on Day 7 (*t* test: *t* = 0.8890, df = 10, *p* = 0.3949) and after withdrawal (*t* test: *t* = 0.5611, df = 10, *p* = 0.5871) in Amph-sensitized male mice compared with saline-treated mice (*t* test: *t* = 4.5808, df = 10, *p* = 0.001). In the same group of male mice, the DAT protein level was significantly reduced following the Amph challenge (*t* test: *t* = 4.5808, df = 10, *p* = 0.001) compared with that level in Amph-sensitized after withdrawal and in saline-treated-sensitized mice. Altogether, the data in Figures 2 and 3 demonstrate that Amph-challenge results in the substantial loss of the axonal DAT protein in sensitized male and not in female mice.

### Amph-induced decrease in HA-DAT level involves endocytic trafficking

**Figure 4.**
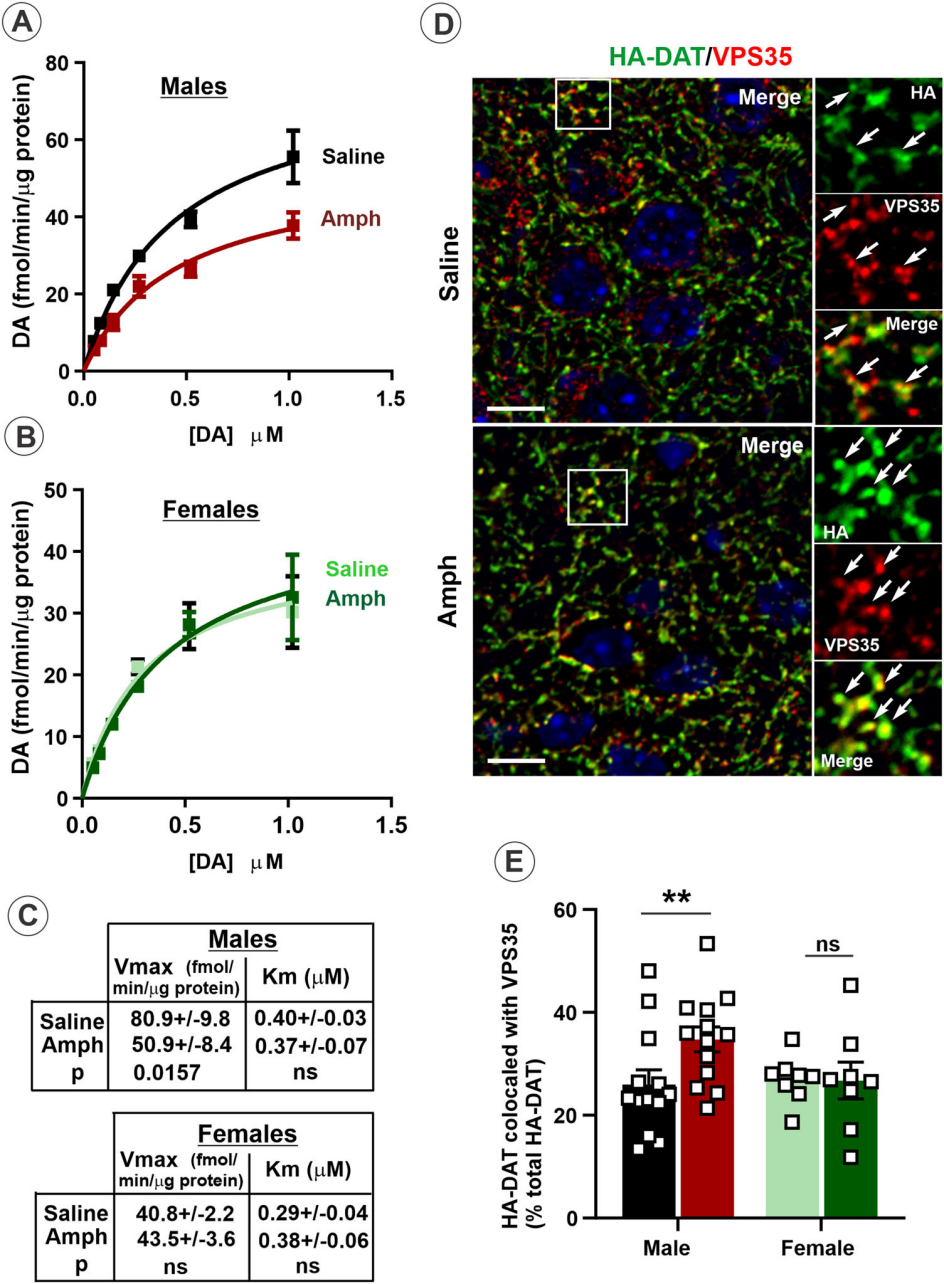
Amph challenge of sensitized male mice leads to reduced DA uptake and increased DAT colocalization with VPS35 in the striatal axons. ***A*–*C***, Kinetic analysis of [^3^H]DA uptake was performed in the striatal synaptosomes of male (***A***) and female (***B***) mice that were challenged with saline or Amph on Day 14. [^3^H]DA (20 nM) and increasing concentrations of unlabeled DA (from 0.005 to 1 µM) were applied simultaneously. The mean values (±SEM) of kinetic parameters (maximal velocity *V*_max_ and affinity constant *K*_m_) derived from experiments exemplified in ***A*,*B*** are presented in ***C***. The data are from three separate experiments, each performed in triplicates. ***D***, Brains were isolated 1 h after saline of AMPH challenge on Day 14 and fixed by cardiac perfusion with 4% paraformaldehyde. Sagittal cryosections were permeabilized and labeled with antibodies against HA (HA-DAT, *green*) and VPS35 (*red*) followed by fluorophore-conjugated secondary antibodies. Nuclei were stained with DAPI (*blue*). 3D images of the striatum were acquired by spinning disk confocal microscope. Maximum-intensity projections of three consecutive confocal sections from the collected z-stacks are shown. Insets show high-magnification images of areas indicated by white rectangles. Arrows point to examples of HA-DAT/VPS35 colocalization in axonal varicosities. Scale bars, 10 µm. ***E***, Quantification of colocalization of HA-DAT and VPS35. Bar graphs show the mean values (±SEM) of the percentage of HA-DAT colocalized with VPS35 of the total HA-DAT fluorescence in 3D images exemplified in ***D***. **p > 0.01; ns, no significant difference (Student's unpaired *t* test). See Extended data Figure 4-1 for quantifications of the expression levels of VPS35 and VPS26 in experiments parallel to those used for quantifications of colocalizations in ***E***.

We and others have previously shown that the bulk of DAT is located at the surface of dopaminergic axons in striatum whereas a pool of intracellular DAT in these axons is extremely small (Nirenberg et al., 1996, 1997a; Block et al., 2015; Bagalkot et al., 2021). Therefore, Amph-induced downregulation of HA-DAT in the striatum demonstrated in Figures 2 and 3 must be associated with the re-distribution of a substantial fraction of DAT from the cell surface via endocytosis to endosomes and/or further to lysosomes. To determine whether Amph challenge of sensitized mice leads to a downregulation of the cell-surface HA-DAT (endocytosis) in a sex-dependent manner, [^3^H]DA uptake assays in synaptosomal preparations were performed, and kinetic parameters were measured (Fig. 4*A–C*). Amph challenge reduced the maximal DA uptake velocity (*V*_max_) by ∼35–45% in Amph-treated males (Amph: *V*_max_ = 50.9 ± 8.4 vs control: *V*_max_ = 80.9 ± 9.8; *p* = 0.0157), whereas no change in the same parameter was observed in experiments with Amph-challenged female mice (Amph: *V*_max_ = 40.8 ± 2.2 vs control: *V*_max_ = 43.5 ± 3.6; *p* = 0.3286) (Fig. 4*A–C*). By contrast, *K*_m_ values of the DA uptake were not significantly different among all experimental variants, suggesting that the sensitization and Amph challenge do not affect the substrate affinity of DAT. Thus, the observation of decreased *V*_max_ is consistent with the hypothesis whereby Amph challenge induces DAT endocytosis leading to a decrease in the DAT concentration at the surface of dopaminergic axons.

10.1523/ENEURO.0491-23.2023.f4-1 Figure 4-1 **Amph challenge does not affect the amounts of retromer complex in sensitized mice.** Striatal synaptosomes (**A**-**B**) and total MB tissue lysates (**C-D**) were electrophoresed and probed by immunoblotting with antibodies to VPS35 (**A** and **C**), VPS26 (**B** and **D**). Representative immunoblots are shown. Bar graphs represent mean values (with SEMs) of VPS35/26 band intensities normalized to TH shown in Figure 2. Quantification of band intensities revealed no significant differences between male and female mice. The data are from three or more independent experiments. ns, no significant difference. Download Figure 4-1, TIF file.

To further investigate the Amph-induced endocytic trafficking process leading to DAT downregulation, we analyzed HA-DAT localization in the striatal axons. We and others recently demonstrated that a pool of axonal DAT is located in endosomes containing VPS35, a subunit of the retromer complex mediating recycling from endosomes, whereas the markers of sorting and late endosomal compartments were undetectable in dopaminergic axons (Block et al., 2015; Wu et al., 2017; Bagalkot et al., 2021). Therefore, co-labeling of HA-DAT and VPS35 was performed on sagittal cryosections of the mouse brain. A substantial colocalization of HA-DAT with VPS35 was observed mainly in axonal varicosities (synaptic areas) in the striatum of both sexes (Fig. 4*D*). Quantifications revealed that Day 14 Amph challenge of sensitized mice resulted in a significant increase of HA-DAT colocalization with VPS35 in the striatal axons in males (*t* test: *t* = 2.3475, df = 24, *p* = 0.0275) but not in females (*t* test: *t* = 0.0598, df = 14, *p* = 0.9532) (Fig. 4*E*). The amount of VPS35 and another retromer complex subunit VPS26 in the striatal synaptosomes and total midbrain tissue homogenates did not significantly change among all experimental variants (Extended data Fig. 4-1), although such measurements do not formally report retromer levels in dopaminergic neurons because these preparations, particularly MB homogenates, contain nondopaminergic cells. Overall, the data in Figure 4*D,E* further support the notion that Amph challenge redistributes a fraction of HA-DAT from the plasma membrane to endosomes in a subset of DA axons in the striatum. Certainly, the diffraction-limited resolution of the confocal microscopy does not allow formal determination of whether HA-DAT immunofluorescence colocalized with VPS35 informs about endosomal HA-DAT given a small size of dopaminergic presynaptic areas. Furthermore, background fluorescence of brain tissue imaging makes the use of the 3D super-resolution imaging and its quantitative analysis to be technically challenging. Nevertheless, together with the observation of Amph-induced decrease in *V*_max_ (Fig. 4*A–C*), increased colocalization of HA-DAT with retromer in males indicates that HA-DAT is internalized and accumulated in early/recycling endosomes after Amph challenge.

**Figure 5.**
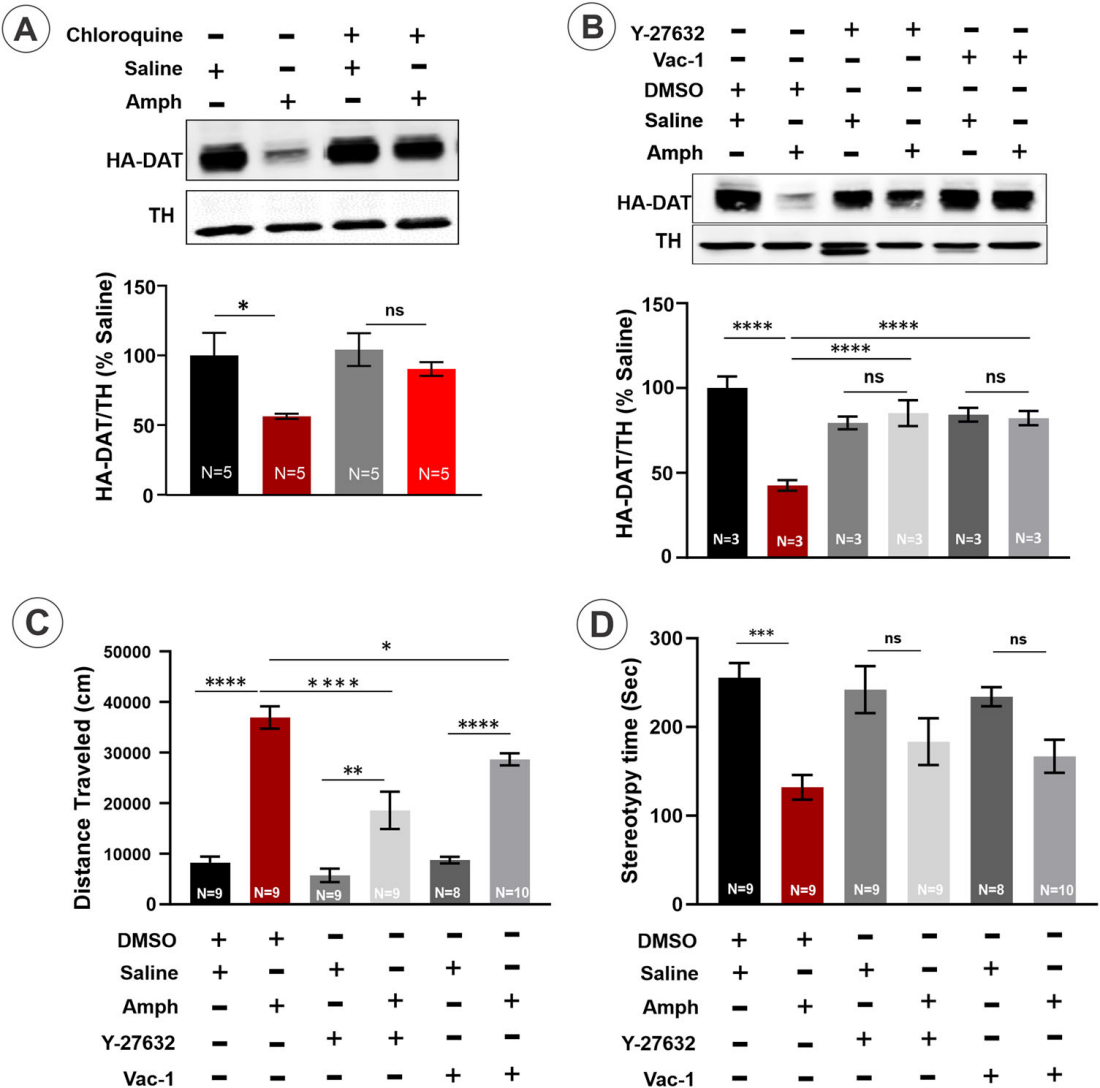
Amph-induced effects on HA-DAT in sensitized mice in the striatum are blunted by inhibitors of endocytosis and endolysosomal traffic. ***A***, Chloroquine (CQ) (100 mg/kg) (“+”) or vehicle (“−” saline) were injected i.p. 2 h before saline or Amph challenge of male mice on Day 14 of the sensitization protocol. Striatal synaptosomes were prepared 1 h after saline/Amph challenge injections, electrophoresed and probed by immunoblotting with antibodies to DAT and TH. Representative immunoblot is shown. Bar graphs show the mean values (±SEM) of the amounts of HA-DAT normalized to the amount of TH and expressed as fractions of the TH-normalized amounts of HA-DAT in “Saline” samples. ***B***, Vehicle (DMSO, “−”), ROCK1/2 inhibitor Y-27632 (5 mg/kg) or vacuolin-1 (*Vac-1*; 2 mg/kg) were injected i.p. 2 h before saline or Amph challenge of male mice on Day 14 of the sensitization protocol. Striatal synaptosomes were prepared 1 h after saline/Amph challenge injections, electrophoresed and probed by immunoblotting with antibodies to DAT and TH. Representative immunoblot is shown. Bar graphs show the mean values (±SEM) of the amounts of HA-DAT normalized to the amount of TH and expressed as fractions of the TH-normalized amounts of HA-DAT in “Saline” samples. ***C*,*D***, On Day 14, sensitized mice were acclimated to individual activity chambers for 1 h before subsequent injections of either Amph (1 mg/kg, i.p.) or saline, 2 h after vehicle (DMSO) and ROCK1/2 inhibitor Y-27632 (5 mg/kg) or vacuolin-1 (2 mg/kg) i.p. injections. Following saline and Amph injections, mice were placed back into their respective activity chambers and their locomotor activity (***C***), and stereotypy time spent (***D***) was recorded. One-way ANOVA, Holm–Sidak's multiple comparisons test, ****p < 0.0001, ***p < 0.001, and *p < 0.05. Error bars represent SEMs. Numbers of animals are indicated on the graph bars.

To examine whether partial loss of HA-DAT protein in the striatal synaptosomes of male mice involves traffic of internalized DAT through the endolysosomal system, Amph-sensitized mice were pretreated with chloroquine (CQ; 100 mg/kg; i.p.). CQ is a membrane-permeable base compound that accumulates in acidic compartments, such as endosomes, autophagosomes, and lysosomes, which results in neutralizing acidic pH in these compartments and blocking endosomal maturation and lysosomal degradation processes. CQ did not influence the amount of HA-DAT in saline-treated mice, but significantly (by ∼46%) diminished Amph-induced HA-DAT loss in the striatal synaptosomes (Amph + CQ group compared with the control “Amph” group) (Fig. 5*A*). These data demonstrate the involvement of DAT trafficking through acidified compartments in the process of apparent Amph-induced DAT degradation.

Further inhibitory analysis was performed to better define the mechanisms of endocytic trafficking of HA-DAT triggered by Amph. Studies by Wheeler and co-workers demonstrated that DAT endocytosis induced by acute Amph requires activation of RhoA GTPase (Wheeler et al., 2015). In the latter study, DAT endocytosis was shown to be sensitive to inhibitors of the main downstream effectors of active RhoA, the Rho-associated coiled-coil containing kinases (ROCK1/2) (Hartmann et al., 2015). Intraperitoneal injection of the ROCK1/2 inhibitor Y-27632 was previously shown to result in ROCK1/2 activity inhibition in mouse brain (Ni et al., 2018). Therefore, we tested the effect of Y-27632 on HA-DAT levels induced by the Amph challenge in male mice and found that this inhibitor dramatically attenuates the Amph effect (Fig. 5*B*). This result suggests that DAT internalization via a RhoA-mediated pathway is involved in the observed DAT protein loss. Moreover, Y-27632 significantly reduced the effect of Amph challenge on the locomotion activity (Fig. 5*C*) without altering the time spent engaged in stereotypy behavior (Fig. 5*D*), thus demonstrating the correlation between Amph-induced decrease in DAT level in the striatum and the behavior response.

**Figure 6.**
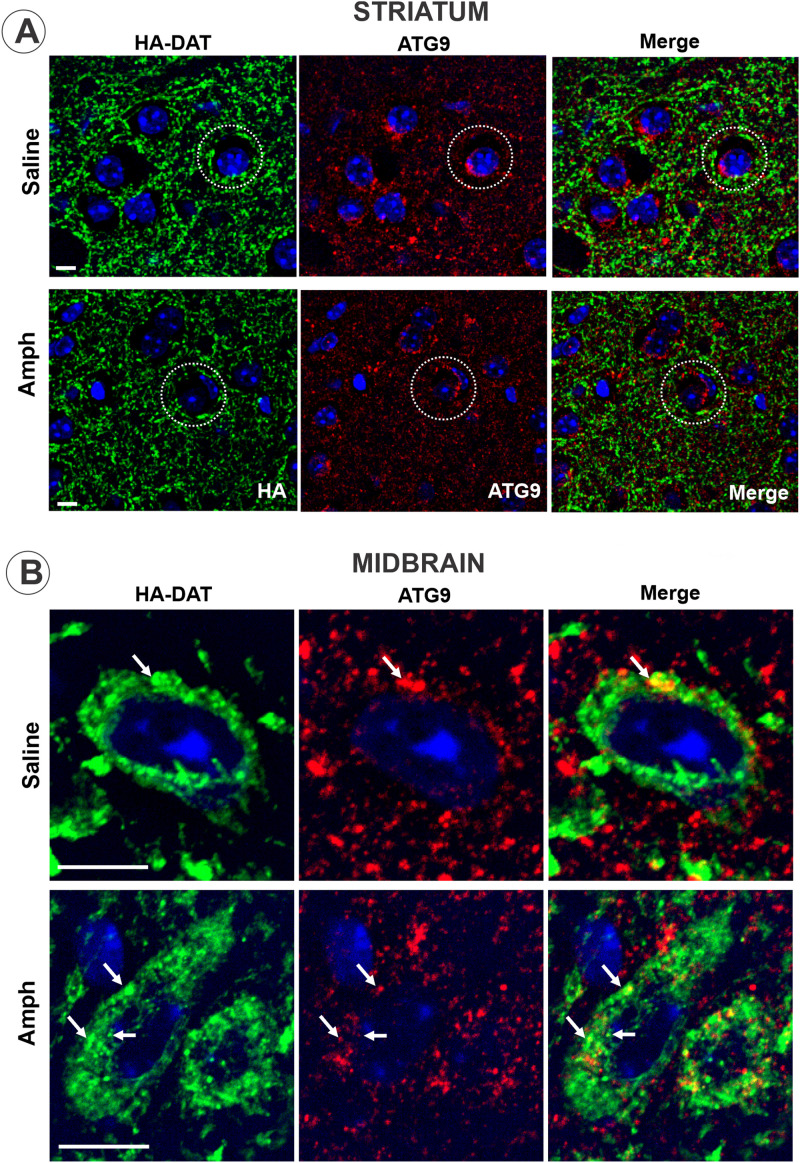
Macroautophagy protein ATG9 is not detected in DA axons in the striatum but is present in the DA neuronal soma. Brains from sensitized and saline- or Amph-challenged male mice were fixed by cardiac perfusion with PFA and prepared for sectioning as described in *Materials and Methods*. Sagittal cryosections were co-labeled with mouse HA11 antibodies and rabbit antibodies against ATG9 (***A*,*B***), followed by fluorophore-conjugated secondary antibodies. 3D confocal images were acquired through 640 nm (red, ATG9), 488 nm (green, HA-DAT), and 405 nm (blue, Hoechst) channels from the striatum (***A***) and midbrain (***B***). White circles represent examples of characteristic perinuclear ATG9 labeling in HA-DAT-negative (nondopaminergic) cells. Arrows show examples of an overlap of ATG9 and HA-DAT fluorescence in neuronal soma. All images are maximum-intensity projections of three consecutive *x–y* confocal sections. Scale bars: ***A*,*B***, 10 µm.

Autophagic degradation of DAT was proposed to underlie the degradation of DAT in dStr after cocaine microinjection (Harraz et al., 2021), although endocytosis of DAT after cocaine injection was not demonstrated. In the latter study, cocaine-induced degradation of DAT was inhibited by vacuolin-1. However, vacuolin-1 has been shown to impose wide-range effects on the endosome–lysosome system through the inhibition of PIKfyve kinase located in early, sorting, and late endosomes, and through the increased activity of Rab5 that blunts endosome maturation (de Lartigue et al., 2009; Lu et al., 2014; Sano et al., 2016). In our experiments, vacuolin-1 also blocked Amph-induced decrease in the HA-DAT protein in the striatum (Fig. 5*B*) and partially decreased Amph-induced locomotor activity (Fig. 5*C*), further supporting the role of post-endocytic traffic of DAT in the effects of Amph challenge in sensitized male mice. While the possibility of autophagocytic processes in dopaminergic synapses has been proposed (Hernandez et al., 2012; Limanaqi et al., 2018; Harraz et al., 2021), the direct evidence (visualization of phagophores or autophagosomes) demonstrating autophagy in presynaptic areas in the intact brain has not been provided. Studies in the intact brain did not detect lysosomal and autophagosomal compartments in dopaminergic axons (Nirenberg et al., 1996, 1997a; Block et al., 2015; Bagalkot et al., 2021). Consistently, the essential macroautophagy protein, ATG9, was not detected by immunofluorescence staining of dopaminergic axons in HA-DAT mice, although it was readily seen in nondopaminergic cells in the striatum and in the soma of dopaminergic neurons (Fig. 6). Therefore, we suggest that upon Amph challenge, striatal DAT traffics through the conventional endocytic pathway rather than undergoing autophagic degradation.

### Amph-induced HA-DAT protein decrease in sensitized male mice is brain-region-specific

**Figure 7.**
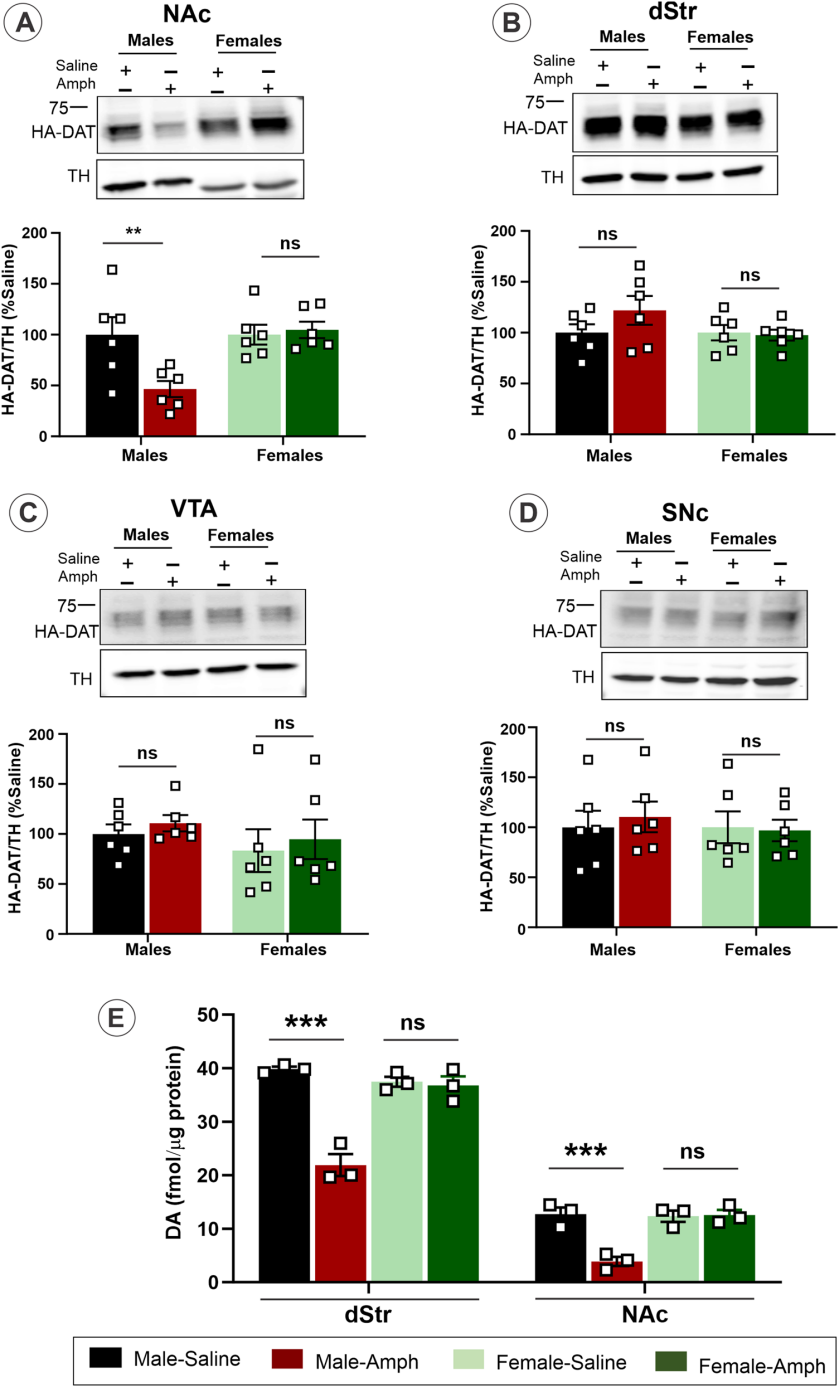
Brain-region-specific decrease in the HA-DAT protein and DA uptake in Amph-sensitized male mice. ***A*–*D***, Tissues from NAc (***A***), dStr (***B***), VTA (***C***), and SNc (***D***) were isolated from sensitized mice challenged with saline or Amph on Day 14. Lysates were electrophoresed and probed by Western blotting with antibodies to DAT and TH. Representative DAT and TH immunoblots are shown. Graph bars show the mean values of the amounts of HA-DAT normalized to the amount of TH and expressed as percent of the mean TH-normalized amounts of HA-DAT in “Saline” samples in each individual experiment. Analysis revealed that HA-DAT protein loss is significant in NAc (*t* test: *t* = 2.8219, df = 10, *p* = 0.0181) (***A***) but not in dStr (*t* test: *t* = 1.3371, df = 10, *p* = 0.2108) (***B***), VTA (*t* test: *t* = 0.8602, df = 10, *p* = 0.4098) (***C***), and SNc (*t* test: *t* = 0.4611, df = 10, *p* = 0.6546) (***D***) in Amph-sensitized male compared with control (“saline”) mice. No significant differences were observed in NAc (*t* test: *t* = 0.3694, df = 10, *p* = 0.7195) (***A***), dStr (*t* test: *t* = 0.2559, df = 10, *p* = 0.8032) (***B***), VTA (*t* test: *t* = 0.1256, df = 10, *p* = 0.9025) (***C***), and SNc (*t* test: *t* = 0.1640, df = 10, *p* = 0.8730) (***D***) in Amph-sensitized female compared with control mice. See Extended data Figure 7-1 for quantifications of the expression level of VPS35 in tissue samples used for HA-DAT quantifications in ***A*–*D***. ***E***, [^3^H]DA uptake was performed in dST and NAc of male and female mice that were challenged with saline or Amph on Day 14. [^3^H]DA (20 nM) and 1 µM of unlabeled DA were applied simultaneously. [^3^H]DA uptake in Amph-sensitized male mice was found to be significantly reduced compared with saline-treated mice in dStr (*t* test: *t* = 8.5966, df = 4, *p* = 0.0010) and NAc (*t* test: *t* = 5.8248, df = 4, *p* = 0.0043) synaptosomes. No difference was observed between saline and Amph-sensitized female mice (dST: *t* test: *t* = 0.3602, df = 4, *p* = 0.7369, NAc: *t* test: *t* = 0.1493, df = 4, *p* = 0.8885). ***p < 0.001; ns, no significant difference (Student's unpaired *t* test).

Our findings of DAT endocytic trafficking triggered by the Amph challenge of sensitized males (Figs. 2–5) are somewhat inconsistent with our previous morphological studies that demonstrated minimal constitutive and acute Amph-induced endocytosis of DAT in dStr (Block et al., 2015). Interestingly, DAT downregulation triggered by the activation of PKC was observed using biotinylation of acute slices from NAc but not dStr, suggesting higher DAT endocytic traffic activity in NAc than in dStr (Fagan et al., 2020; Sweeney et al., 2020). The dStr and NAc are technically difficult to precisely localize using fluorescence microscopy within a sagittal brain section. Therefore, to test whether Amph effects on HA-DAT in sensitized mice are striatum-region-specific, an immunoblotting analysis of HA-DAT in total tissue lysates of NAc, dST, VTA, and SNc isolated from saline- or Amph-challenged sensitized mice was performed. This analysis revealed a significant Amph-induced HA-DAT loss in NAc (*t* test: *t* = 2.8219, df =10, *p* = 0.0181) (Fig. 7*A*) but not in dStr of males (Fig. 7*B*). No changes in HA-DAT levels were observed in VTA (Fig. 7*C*) and SNc (Fig. 7*D*) recovered from male mice. No significant differences in the amount of HA-DAT in Amph-sensitized compared with control mice were observed in either of the brain areas of female mice (Fig. 7*A–D*). The immunoblotting did not reveal changes in the amount of VPS35 in lysates of NAc, dStr, VTA, and SNc in sensitized mice of either sex treated with saline versus Amph (Extended data Fig. 7-1).

10.1523/ENEURO.0491-23.2023.f7-1 Figure 7-1 **Amph challenge does not affect VPS35 levels in all brain regions tested in sensitized mice.** Tissue lysates from NAc (**A**), dStr (**B**), VTA (**C**), and SNc (**D**) were electrophoresed and probed by immunoblotting with antibodies to VPS35 (**A-D**). Representative immunoblots are shown. Bar graphs represent mean values (with SEMs) of VPS35 band intensities normalized to TH. Quantification of band intensities revealed no significant differences between male and female mice. The data are from three or more independent experiments. ns, no significant difference. Download Figure 7-1, TIF file.

The observation of the absence of Amph-induced loss of HA-DAT in dStr is somewhat inconsistent with the significant endocytic trafficking demonstrated by increased VPS35 colocalization and decreased *V*_max_ of the DA uptake in synaptosomes prepared from the entire striatum (Fig. 4), given that dStr is the largest region of the striatum within sagittal brain sections and the main source of synaptosomes. Therefore, [^3^H]DA uptake measurements at saturating DA concentration (1 mM) were performed to compare the maximum uptake capacity in dST and ventral striatum (containing NAc) synaptosomes to assess region-specific downregulation of cell-surface HA-DAT. These experiments revealed that the DA uptake in Amph-sensitized male mice is significantly reduced compared with saline-treated mice in both dST (*t* test: *t* = 8.5966, df = 4, *p* = 0.0010) and ventral striatum (*t* test: *t* = 5.8248, df = 4, *p* = 0.0043) synaptosomes (Fig. 7*E*). No difference was observed between saline and Amph-sensitized female mice (dST: *t* test: *t* = 0.3602, df = 4, *p* = 0.7369; NAc: *t* test: *t* = 0.1493, df = 4, *p* = 0.8885) (Fig. 7*E*). Collectively, the data in Figure 7 indicate that while Amph challenge on Day 14 triggers downregulation of cell-surface HA-DAT in both dStr and NAc, the sex-specific loss of DAT is observed only in NAc.

## Discussion

In this study, we have used several complementary approaches to show that a short Amph challenge of Amph-sensitized mice results in endocytosis (internalization) and a substantial reduction in the amount of DAT in the striatum in male, but not in female mice. This DAT protein loss in sensitized male mice was on average by 30–60% as shown by immunoblotting of striatal synaptosomes in Figures 2, 3, and 5 and Extended data Figure 2-1. Surprisingly, this apparent degradation of DAT was observed in NAc, and not in dStr (Fig. 7). At the same time, Amph challenge reduced the maximal DA uptake capacity in both dStr and ventral striatum, indicative of DAT endocytosis in both regions of the striatum. The demonstration of the involvement of RhoA signaling (using ROCK inhibitor) in the Amph-induced partial loss of DAT using the synaptosome-based assay also supports the notion that DAT endocytosis is triggered by Amph in dStr, the major source of synaptosomes, as well as in NAc. By contrast, the lack of detectable Amph-induced decrease in the DAT levels in the midbrain tissue was not unexpected provided that a bulk of DAT has been shown to be located intracellularly in the somatodendritic compartment of dopaminergic neurons, mostly in the endoplasmic reticulum and Golgi complex (Nirenberg et al., 1996, 1997b; Block et al., 2015; Bagalkot et al., 2021) (also Fig. 6).

Altogether, the data prompt us to propose a model whereby Amph challenge of sensitized male mice results in DAT endocytosis in both dStr and NAc, whereas an apparent degradation of internalized DAT protein occurs mainly in NAc. Whether a fraction of DAT is indeed degraded within 1 h after the Amph administration in NAc axons or transported out of NAc followed by the distribution throughout the medial forebrain bundle (MFB) axons and somatodendritic compartment of neurons remains unclear. Against the first possibility is the lack of degradative compartments (lysosomes) in the striatal dopaminergic axons as shown by immunofluorescence and electron microscopy studies (Nirenberg et al., 1996, 1997a; Block et al., 2015; Bagalkot et al., 2021), with the caveat that morphological approaches may not be sufficiently sensitive to formally rule out the presence of a small number of lysosomes in these axons, especially in a less studied NAc. The anterograde transport of lysosome-like organelles to axons of cultured hippocampal neurons was shown to be mediated by Arl8 GTPase and a multi-subunit complex named BORC (Guardia et al., 2016; Keren-Kaplan and Bonifacino, 2021). Thus, enhancing the activity of these and/or other regulatory components of the anterograde lysosome transport may theoretically be the potential mechanism for accumulation of lysosomes in NAc in response to stimuli such as amphetamine in sensitized mice. However, we are not aware of the published data demonstrating anterograde transport of lysosomes to dopaminergic axons or its regulation in adult rodent brain. The second model would entail rapid microtubular-mediated transport of DAT in endosomes to the neuronal soma where DAT can be degraded in lysosomes. Our previous analysis of the long-range transport of DAT between the striatum and midbrain through MFB demonstrated the prevalence of a slower transport mechanism, lateral membrane diffusion (Bagalkot et al., 2021). This makes it to be unlikely that a significant number of DAT molecules can be transported through MFB within 1 h of Amph challenge to allow their lysosomal degradation in the soma. However, in the latter studies, we have also detected a subset of MFB axons in which a significant amount of DAT was present in VPS35-positive endocytic vesicles (Bagalkot et al., 2021), suggesting that a fraction of DAT can be transported through MFB in endocytic vesicles using a faster microtubular-dependent mechanism.

While the behavior effects of Amph are the result of increased DA release and DAT-mediated DA efflux in addition to DAT endocytosis, different kinetics of Amph-induced locomotor activity in males and females (Fig. 1*F,J*) correlate and are consistent with the male-specific endocytic downregulation of DAT in sensitized mice (Figs. 2–5, 7). In females, surface DAT is not downregulated, and therefore, an excess of DA is removed, which results in a decrease in the locomotor activity after initial temporary increase. In males, cell-surface DAT is downregulated, extracellular DA is not efficiently removed, and therefore, a high locomotor activity is sustained. The observation of a strong effect of the ROCK1/2 inhibitor on the behavior response to Amph is in agreement with the model that Amph induces RhoA-dependent endocytosis of DAT (Wheeler et al., 2015), and therefore reduces DAT density at the cell surface and DA reuptake. That an endocytosis step of DAT trafficking can be regulated in a sex-specific manner was demonstrated by sex-dependent effects of the depletion of Rit2, a small GTPase known to regulate PKC-induced DAT endocytosis, on DAT distribution in dopaminergic neurons (Fagan et al., 2020; Sweeney et al., 2020). The impact of the inhibition of PIKfyve kinase by vacuolin-1 on the behavior response to Amph challenge was relatively small (Fig. 5). Since vacuolin-1 affects the endolysosomal system, it may alter post-endocytic DAT traffic, including DAT sorting to lysosomes and recycling from endosomes to the plasma membrane. However, vacuolin-1 is not expected to block DAT internalization and significantly decrease the amount of DAT at the cell surface.

Our finding of sex-dependent effects of 1 h challenge Amph on DAT and locomotion activity in sensitized mice was unexpected. Most of the previous studies in rodents show a more robust behavioral sensitization of females than males in response to single or repeated injections of Amph or methamphetamine (Robinson, 1984), although there have been reports of a greater Amph-induced sensitization in males (Flemenbaum, 1979). However, comparison of these studies with our results demonstrating that female mice do not exhibit a sustained Amph-induced locomotor sensitization is not straightforward. First, much higher doses of Amph compared with our experiments, which may lead to stereotypical behaviors (Shen et al., 2001; Chinen et al., 2006), have been used in previous studies (Robinson, 1984). Second, many studies performed 30 min tests (Hyde and Jerussi, 1983; Mathews and McCormick, 2007; Davis et al., 2022) that may have precluded the detection of different behavior kinetics in males and females observed in our experiments after 30 min of Amph challenge. The mechanisms underlying sex-dependence of Amph effects on DAT observed in our studies are unclear. We have not observed differences in the DAT protein concentration and DA uptake in the striatum of males versus females in untreated brains (Figs. 2–5, 7). By contrast, a fast-scan cyclic voltammetry study found that the DA reuptake in the dorsolateral striatum is greater in rat females than males (Walker et al., 2000). Ovary removal was demonstrated to upregulate DAT in the absence of estradiol (Attali et al., 1997), which indicates that DAT levels/traffic may be regulated by estrogen receptors in female rodents. Overall, the role of sex in DAT regulation has been sparsely studied. Our studies are limited by measuring DAT levels, DA uptake, and VPS35:DAT colocalization 1 h after Amph treatment on Day 14. Certainly, analyses of the temporal dynamics of DAT endocytosis at earlier days of the sensitization schedule and at multiple time points within 1 h after Amph challenge can be instrumental in dissecting the mechanisms of underlying sex-dependent DAT trafficking. Future efforts in the elucidation of the molecular mechanisms of Amph-induced DAT trafficking in sensitized animals and its sex-dependence would require the development of new technical approaches allowing high spatial and temporal resolution analysis of DAT trafficking in intact mouse brain.

## Synthesis

Reviewing Editor: Michael Michaelides, NIDA-NIH Decisions are customarily a result of the Reviewing Editor and the peer reviewers coming together and discussing their recommendations until a consensus is reached. When revisions are invited, a fact-based synthesis statement explaining their decision and outlining what is needed to prepare a revision will be listed below. The following reviewer(s) agreed to reveal their identity: Habibeh Khoshbouei. Note: If this manuscript was transferred from JNeurosci and a decision was made to accept the manuscript without peer review, a brief statement to this effect will instead be what is listed below.

Reviewer 1

Thanks for the careful revision of the manuscript. The revisions have addressed this reviewer's concern.

## Author Response

Following concerns were addressed in the revised manuscript:

1. The term "complimentary" was corrected as "complementary" in the Discussion. This change is highlighted in yellow.

2. The position of the label "Amph" in the bottom panel of Figure 6 was corrected.

## References

[B1] Attali G, Weizman A, Gil-Ad I, Rehavi M (1997) Opposite modulatory effects of ovarian hormones on rat brain dopamine and serotonin transporters. Brain Res 756:153–159. 10.1016/S0006-8993(97)00136-4 9187326

[B2] Bagalkot TR, Block ER, Bucchin K, Balcita-Pedicino JJ, Calderon M, Sesack SR, Sorkin A (2021) Dopamine transporter localization in medial forebrain bundle axons indicates its long-range transport primarily by membrane diffusion with a limited contribution of vesicular traffic on retromer-positive compartments. J Neurosci 41:234–250. 10.1523/JNEUROSCI.0744-20.2020 33234607 PMC7810657

[B3] Becker JB, Molenda H, Hummer DL (2001) Gender differences in the behavioral responses to cocaine and amphetamine. Implications for mechanisms mediating gender differences in drug abuse. Ann N Y Acad Sci 937:172–187. 10.1111/j.1749-6632.2001.tb03564.x 11458536

[B4] Block ER, Nuttle J, Balcita-Pedicino JJ, Caltagarone J, Watkins SC, Sesack SR, Sorkin A (2015) Brain region-specific trafficking of the dopamine transporter. J Neurosci 35:12845–12858. 10.1523/JNEUROSCI.1391-15.2015 26377471 PMC4571607

[B5] Boileau I, Dagher A, Leyton M, Gunn RN, Baker GB, Diksic M, Benkelfat C (2006) Modeling sensitization to stimulants in humans: an [11C]raclopride/positron emission tomography study in healthy men. Arch Gen Psychiatry 63:1386–1395. 10.1001/archpsyc.63.12.1386 17146013

[B6] Booij L, et al. (2016) Dopamine cross-sensitization between psychostimulant drugs and stress in healthy male volunteers. Transl Psychiatry 6:e740. 10.1038/tp.2016.6 26905412 PMC4872435

[B7] Bourque M, Liu B, Dluzen DE, Di Paolo T (2011) Sex differences in methamphetamine toxicity in mice: effect on brain dopamine signaling pathways. Psychoneuroendocrinology 36:955–969. 10.1016/j.psyneuen.2010.12.007 21236583

[B8] Chen R, Furman CA, Zhang M, Kim MN, Gereau RWt, Leitges M, Gnegy ME (2009) Protein kinase Cbeta is a critical regulator of dopamine transporter trafficking and regulates the behavioral response to amphetamine in mice. J Pharmacol Exp Ther 328:912–920. 10.1124/jpet.108.147959 19098163 PMC2682265

[B9] Chi L, Reith ME (2003) Substrate-induced trafficking of the dopamine transporter in heterologously expressing cells and in rat striatal synaptosomal preparations. J Pharmacol Exp Ther 307:729–736. 10.1124/jpet.103.055095 12975490

[B10] Chinen CC, Faria RR, Frussa-Filho R (2006) Characterization of the rapid-onset type of behavioral sensitization to amphetamine in mice: role of drug-environment conditioning. Neuropsychopharmacology 31:151–159. 10.1038/sj.npp.1300789 15956986

[B11] Dani JA, Zhou FM (2004) Selective dopamine filter of glutamate striatal afferents. Neuron 42:522–524. 10.1016/j.neuron.2004.05.008 15157413

[B12] Davis DL, Metzger DB, Vann PH, Wong JM, Subasinghe KH, Garlotte IK, Phillips NR, Shetty RA, Forster MJ, Sumien N (2022) Sex differences in neurobehavioral consequences of methamphetamine exposure in adult mice. Psychopharmacology 239:2331–2349. 10.1007/s00213-022-06122-8 35347365 PMC9232998

[B13] de Lartigue J, Polson H, Feldman M, Shokat K, Tooze SA, Urbe S, Clague MJ (2009) PIKfyve regulation of endosome-linked pathways. Traffic 10:883–893. 10.1111/j.1600-0854.2009.00915.x 19582903 PMC2723830

[B14] Dluzen DE, McDermott JL (2008) Sex differences in dopamine- and vesicular monoamine-transporter functions. Ann N Y Acad Sci 1139:140–150. 10.1196/annals.1432.010 18991858

[B15] Fagan RR, Kearney PJ, Sweeney CG, Luethi D, Schoot Uiterkamp FE, Schicker K, Alejandro BS, O'Connor LC, Sitte HH, Melikian HE (2020) Dopamine transporter trafficking and Rit2 GTPase: mechanism of action and in vivo impact. J Biol Chem 295:5229–5244. 10.1074/jbc.RA120.012628 32132171 PMC7170531

[B16] Flemenbaum A (1979) Rat dopaminergic hypersensitivity. II. Effects of sex. Neuropsychobiology 5:222–231. 10.1159/000117686 571545

[B17] Gainetdinov RR, Caron MG (2003) Monoamine transporters: from genes to behavior. Annu Rev Pharmacol Toxicol 43:261–284. 10.1146/annurev.pharmtox.43.050802.112309 12359863

[B18] Garcia BG, Wei Y, Moron JA, Lin RZ, Javitch JA, Galli A (2005) Akt is essential for insulin modulation of amphetamine-induced human dopamine transporter cell-surface redistribution. Mol Pharmacol 68:102–109. 10.1124/mol.104.009092 15795321

[B19] Gatica RI, Aguilar-Rivera MI, Azocar VH, Fuentealba JA (2020) Individual differences in amphetamine locomotor sensitization are accompanied with changes in dopamine release and firing pattern in the dorsolateral striatum of rats. Neuroscience 427:116–126. 10.1016/j.neuroscience.2019.11.048 31874242

[B20] German CL, Hanson GR, Fleckenstein AE (2012) Amphetamine and methamphetamine reduce striatal dopamine transporter function without concurrent dopamine transporter relocalization. J Neurochem 123:288–297. 10.1111/j.1471-4159.2012.07875.x 22804716 PMC3962019

[B21] Giros B, Caron MG (1993) Molecular characterization of the dopamine transporter. Trends Pharmacol Sci 14:43–49. 10.1016/0165-6147(93)90029-J 8480373

[B22] Gowrishankar R, Hahn MK, Blakely RD (2014) Good riddance to dopamine: roles for the dopamine transporter in synaptic function and dopamine-associated brain disorders. Neurochem Int 73:42–48. 10.1016/j.neuint.2013.10.016 24231471

[B23] Guardia CM, Farias GG, Jia R, Pu J, Bonifacino JS (2016) BORC functions upstream of kinesins 1 and 3 to coordinate regional movement of lysosomes along different microtubule tracks. Cell Rep 17:1950–1961. 10.1016/j.celrep.2016.10.062 27851960 PMC5136296

[B24] Harraz MM, et al. (2021) Cocaine-induced locomotor stimulation involves autophagic degradation of the dopamine transporter. Mol Psychiatry 26:370–382. 10.1038/s41380-020-00978-y 33414501 PMC8625012

[B25] Hartmann S, Ridley AJ, Lutz S (2015) The function of Rho-associated kinases ROCK1 and ROCK2 in the pathogenesis of cardiovascular disease. Front Pharmacol 6:276. 10.3389/fphar.2015.00276 26635606 PMC4653301

[B26] Hernandez D, et al. (2012) Regulation of presynaptic neurotransmission by macroautophagy. Neuron 74:277–284. 10.1016/j.neuron.2012.02.020 22542182 PMC3578406

[B27] Hong WC, Amara SG (2013) Differential targeting of the dopamine transporter to recycling or degradative pathways during amphetamine- or PKC-regulated endocytosis in dopamine neurons. FASEB J 27:2995–3007. 10.1096/fj.12-218727 23612789 PMC3714572

[B28] Hyde JF, Jerussi TP (1983) Sexual dimorphism in rats with respect to locomotor activity and circling behavior. Pharmacol Biochem Behav 18:725–729. 10.1016/0091-3057(83)90014-X 6856647

[B29] Iversen SD, Iversen LL (2007) Dopamine: 50 years in perspective. Trends Neurosci 30:188–193. 10.1016/j.tins.2007.03.002 17368565

[B30] Johnson LA, Furman CA, Zhang M, Guptaroy B, Gnegy ME (2005) Rapid delivery of the dopamine transporter to the plasmalemmal membrane upon amphetamine stimulation. Neuropharmacology 49:750–758. 10.1016/j.neuropharm.2005.08.018 16212991

[B31] Kahlig KM, Javitch JA, Galli A (2004) Amphetamine regulation of dopamine transport. Combined measurements of transporter currents and transporter imaging support the endocytosis of an active carrier. J Biol Chem 279:8966–8975. 10.1074/jbc.M303976200 14699142

[B32] Kalivas PW, Duffy P (1993) Time course of extracellular dopamine and behavioral sensitization to cocaine. II. Dopamine perikarya. J Neurosci 13:276–284. 10.1523/JNEUROSCI.13-01-00276.1993 8380850 PMC6576314

[B33] Keren-Kaplan T, Bonifacino JS (2021) ARL8 relieves SKIP autoinhibition to enable coupling of lysosomes to kinesin-1. Curr Biol 31:540–554.e5. 10.1016/j.cub.2020.10.071 33232665 PMC7878431

[B34] Khoshbouei H, Wang H, Lechleiter JD, Javitch JA, Galli A (2003) Amphetamine-induced dopamine efflux. A voltage-sensitive and intracellular Na+-dependent mechanism. J Biol Chem 278:12070–12077. 10.1074/jbc.M212815200 12556446

[B35] Limanaqi F, Biagioni F, Gambardella S, Ryskalin L, Fornai F (2018) Interdependency between autophagy and synaptic vesicle trafficking: implications for dopamine release. Front Mol Neurosci 11:299. 10.3389/fnmol.2018.00299 30186112 PMC6110820

[B36] Lu Y, et al. (2014) Vacuolin-1 potently and reversibly inhibits autophagosome-lysosome fusion by activating RAB5A. Autophagy 10:1895–1905. 10.4161/auto.32200 25483964 PMC4502727

[B37] Mathews IZ, McCormick CM (2007) Female and male rats in late adolescence differ from adults in amphetamine-induced locomotor activity, but not in conditioned place preference for amphetamine. Behav Pharmacol 18:641–650. 10.1097/FBP.0b013e3282effbf5 17912048

[B38] Ni M, Zhang J, Huang L, Liu G, Li Q (2018) A Rho-kinase inhibitor reverses learning and memory deficits in a rat model of chronic cerebral ischemia by altering Bcl-2/Bax-NMDAR signaling in the cerebral cortex. J Pharmacol Sci 138:107–115. 10.1016/j.jphs.2018.08.012 30366873

[B39] Nirenberg M, Vaughan R, Uhl G, Kuhar M, Pickel V (1996) The dopamine transporter is localized to dendritic and axonal plasma membranes of nigrostriatal dopaminergic neurons. J Neurosci 16:436–447. 10.1523/JNEUROSCI.16-02-00436.1996 8551328 PMC6578661

[B40] Nirenberg MJ, Chan J, Pohorille A, Vaughan RA, Uhl GR, Kuhar MJ, Pickel VM (1997a) The dopamine transporter: comparative ultrastructure of dopaminergic axons in limbic and motor compartments of the nucleus accumbens. J Neurosci 17:6899–6907. 10.1523/JNEUROSCI.17-18-06899.1997 9278525 PMC6573281

[B41] Nirenberg MJ, Chan J, Vaughan RA, Uhl GR, Kuhar MJ, Pickel VM (1997b) Immunogold localization of the dopamine transporter: an ultrastructural study of the rat ventral tegmental area. J Neurosci 17:5255–5262. 10.1523/JNEUROSCI.17-14-05255.1997 9204909 PMC6793826

[B42] Paulson PE, Robinson TE (1995) Amphetamine-induced time-dependent sensitization of dopamine neurotransmission in the dorsal and ventral striatum: a microdialysis study in behaving rats. Synapse 19:56–65. 10.1002/syn.890190108 7709344 PMC1859849

[B43] Pierce RC, Kalivas PW (1995) Amphetamine produces sensitized increases in locomotion and extracellular dopamine preferentially in the nucleus accumbens shell of rats administered repeated cocaine. J Pharmacol Exp Ther 275:1019–1029. https://www.ncbi.nlm.nih.gov/pubmed/7473128 7473128

[B44] Rao A, Richards TL, Simmons D, Zahniser NR, Sorkin A (2012) Epitope-tagged dopamine transporter knock-in mice reveal rapid endocytic trafficking and filopodia targeting of the transporter in dopaminergic axons. FASEB J 26:1921–1933. 10.1096/fj.11-196113 22267337 PMC3336793

[B45] Rao A, Sorkin A, Zahniser NR (2013) Mice expressing markedly reduced striatal dopamine transporters exhibit increased locomotor activity, dopamine uptake turnover rate, and cocaine responsiveness. Synapse 67:668–677. 10.1002/syn.21671 23564231 PMC3760678

[B46] Richards TL, Zahniser NR (2009) Rapid substrate-induced down-regulation in function and surface localization of dopamine transporters: rat dorsal striatum versus nucleus accumbens. J Neurochem 108:1575–1584. 10.1111/j.1471-4159.2009.05910.x 19183252 PMC2885765

[B47] Robertson SD, Matthies HJ, Galli A (2009) A closer look at amphetamine-induced reverse transport and trafficking of the dopamine and norepinephrine transporters. Mol Neurobiol 39:73–80. 10.1007/s12035-009-8053-4 19199083 PMC2729543

[B48] Robinson TE (1984) Behavioral sensitization: characterization of enduring changes in rotational behavior produced by intermittent injections of amphetamine in male and female rats. Psychopharmacology 84:466–475. 10.1007/BF00431451 6441946

[B49] Robinson TE, Badiani A (1998) Drug-induced adaptations in catecholamine systems: on the inevitability of sensitization. Adv Pharmacol 42:987–990. 10.1016/S1054-3589(08)60912-6 9328063

[B50] Robinson TE, Becker JB (1986) Enduring changes in brain and behavior produced by chronic amphetamine administration: a review and evaluation of animal models of amphetamine psychosis. Brain Res 396:157–198. 10.1016/0165-0173(86)90002-0 3527341

[B51] Robinson TE, Browman KE, Crombag HS, Badiani A (1998) Modulation of the induction or expression of psychostimulant sensitization by the circumstances surrounding drug administration. Neurosci Biobehav Rev 22:347–354. 10.1016/S0149-7634(97)00020-1 9579324

[B52] Sano O, Kazetani K, Funata M, Fukuda Y, Matsui J, Iwata H (2016) Vacuolin-1 inhibits autophagy by impairing lysosomal maturation via PIKfyve inhibition. FEBS Lett 590:1576–1585. 10.1002/1873-3468.12195 27135648

[B53] Saunders C, Ferrer JV, Shi L, Chen J, Merrill G, Lamb ME, Leeb-Lundberg LM, Carvelli L, Javitch JA, Galli A (2000) Amphetamine-induced loss of human dopamine transporter activity: an internalization-dependent and cocaine-sensitive mechanism. Proc Natl Acad Sci U S A 97:6850–6855. 10.1073/pnas.110035297 10823899 PMC18764

[B54] Schultz W (2002) Getting formal with dopamine and reward. Neuron 36:241–263. 10.1016/S0896-6273(02)00967-4 12383780

[B55] Segal DS, Kuczenski R (1992) In vivo microdialysis reveals a diminished amphetamine-induced DA response corresponding to behavioral sensitization produced by repeated amphetamine pretreatment. Brain Res 571:330–337. 10.1016/0006-8993(92)90672-V 1377088

[B56] Shen TF, Wang HC, Wan FJ, Tung CS (2001) Changes in the performance of schedule-induced polydipsia (SIP) in rats after arecoline and amphetamine treatments. Proc Natl Sci Counc Repub China B 25:214–222. https://www.ncbi.nlm.nih.gov/pubmed/11699569 11699569

[B57] Snyder SH (2002) Forty years of neurotransmitters: a personal account. Arch Gen Psychiatry 59:983–994. 10.1001/archpsyc.59.11.983 12418931

[B58] Sorkina T, Doolen S, Galperin E, Zahniser NR, Sorkin A (2003) Oligomerization of dopamine transporters visualized in living cells by fluorescence resonance energy transfer microscopy. J Biol Chem 278:28274–28283. 10.1074/jbc.M210652200 12746456

[B59] Sorkina T, Ma S, Larsen MB, Watkins SC, Sorkin A (2018) Small molecule induced oligomerization, clustering and clathrin-independent endocytosis of the dopamine transporter. Elife 7:e32293. 10.7554/eLife.32293 29630493 PMC5896956

[B60] Sulzer D, Sonders MS, Poulsen NW, Galli A (2005) Mechanisms of neurotransmitter release by amphetamines: a review. Prog Neurobiol 75:406–433. 10.1016/j.pneurobio.2005.04.003 15955613

[B61] Sweeney CG, et al. (2020) Conditional, inducible gene silencing in dopamine neurons reveals a sex-specific role for Rit2 GTPase in acute cocaine response and striatal function. Neuropsychopharmacology 45:384–393. 10.1038/s41386-019-0457-x 31277075 PMC6901441

[B62] Volkow ND, Morales M (2015) The brain on drugs: from reward to addiction. Cell 162:712–725. 10.1016/j.cell.2015.07.046 26276628

[B63] Walker QD, Rooney MB, Wightman RM, Kuhn CM (2000) Dopamine release and uptake are greater in female than male rat striatum as measured by fast cyclic voltammetry. Neuroscience 95:1061–1070. 10.1016/S0306-4522(99)00500-X 10682713

[B64] Wheeler DS, Underhill SM, Stolz DB, Murdoch GH, Thiels E, Romero G, Amara SG (2015) Amphetamine activates Rho GTPase signaling to mediate dopamine transporter internalization and acute behavioral effects of amphetamine. Proc Natl Acad Sci U S A 112:E7138–7147. 10.1073/pnas.1511670112 26553986 PMC4697400

[B65] Wise RA (2008) Dopamine and reward: the anhedonia hypothesis 30 years on. Neurotox Res 14:169–183. 10.1007/BF03033808 19073424 PMC3155128

[B66] Wolf ME, White FJ, Nassar R, Brooderson RJ, Khansa MR (1993) Differential development of autoreceptor subsensitivity and enhanced dopamine release during amphetamine sensitization. J Pharmacol Exp Ther 264:249–255. https://www.ncbi.nlm.nih.gov/pubmed/8093727 8093727

[B67] Wu S, Fagan RR, Uttamapinant C, Lifshitz LM, Fogarty KE, Ting AY, Melikian HE (2017) The dopamine transporter recycles via a retromer-dependent postendocytic mechanism: tracking studies using a novel fluorophore-coupling approach. J Neurosci 37:9438–9452. 10.1523/JNEUROSCI.3885-16.2017 28847807 PMC5618262

