# Supplementary figures and images for: Amphetamine Induces Sex-Dependent Loss of the Striatal Dopamine Transporter in Sensitized Mice
Source: eNeuro. 2024 Jan 10;11(1):ENEURO.0491-23.2023. doi: 10.1523/ENEURO.0491-23.2023 (PMC10849026; doi:10.1523/ENEURO.0491-23.2023)

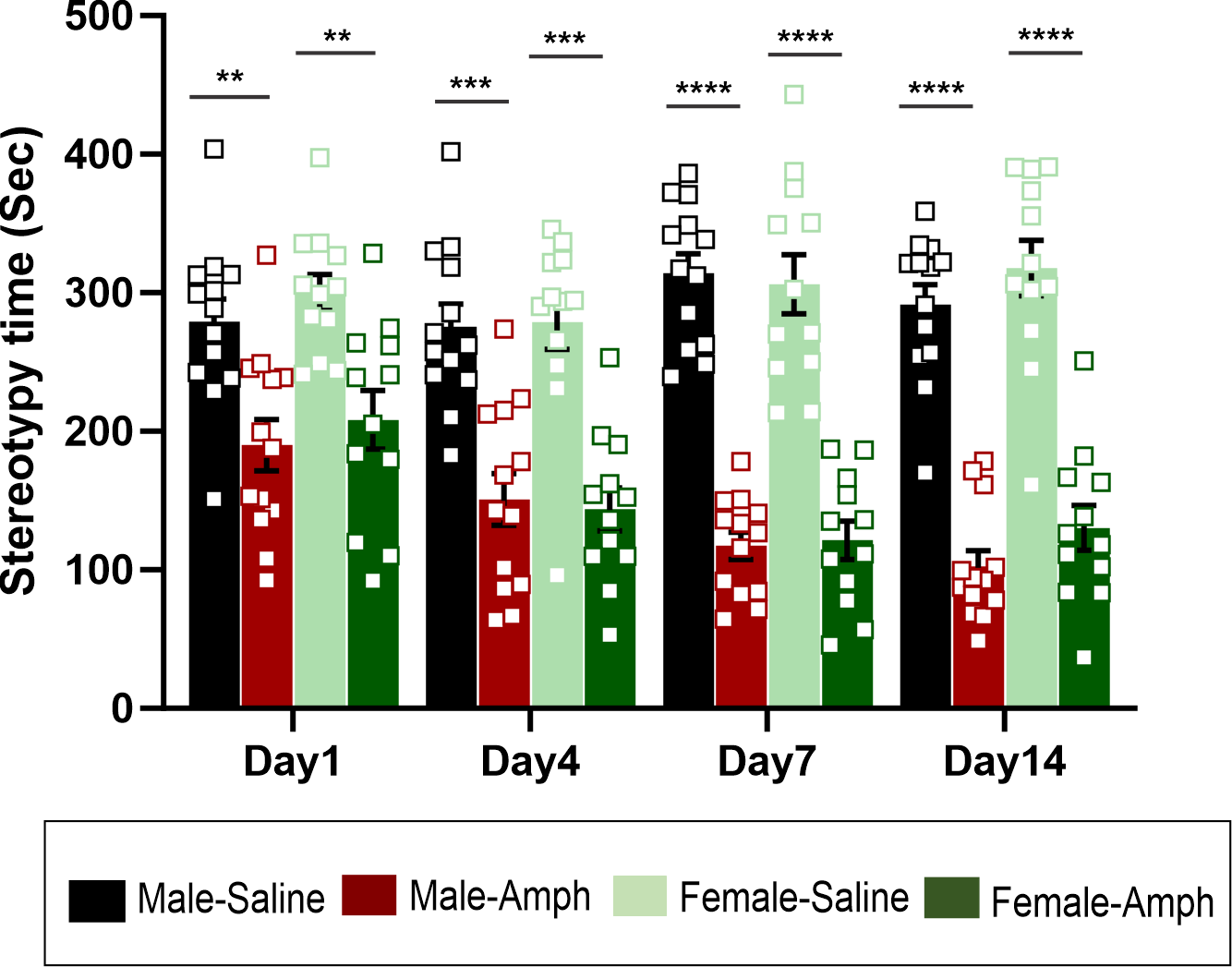

Supplement: Figure 1-1 — Repeated administration of Amph results in decreased in time spent showing stereotypy-like behavior in sensitized mice. Stereotypy time was measured for 1 hr after saline or Amph injection on day 1, 4, 7 and after Amph challenge on day 14. Two-way repeated measures ANOVA analysis showed a significant main effect of group (F3,46 = 58.06, p˂0.0001), day (F2.911, 133.9= 5.398, p˂0.001), and interaction of group x day (F9,138= 4.571, p˂0.0001). Tukey’s post hoc analysis revealed decreased stereotypy time spent on day 1, 4, 7 and 14 of AMPH treated male and female mice compared to respective saline groups (Male: Day 1; p=0.008, Day 4; p˂0.000, Day 7; p˂0.001 and Day 14; p˂0.0001, and Female: p=0.008, p˂0.0001, p˂0.0001 and p˂0.0001, respectively). Download Figure 1-1, TIF file. [file eneuro-11-ENEURO.0491-23.2023-s001.tif]

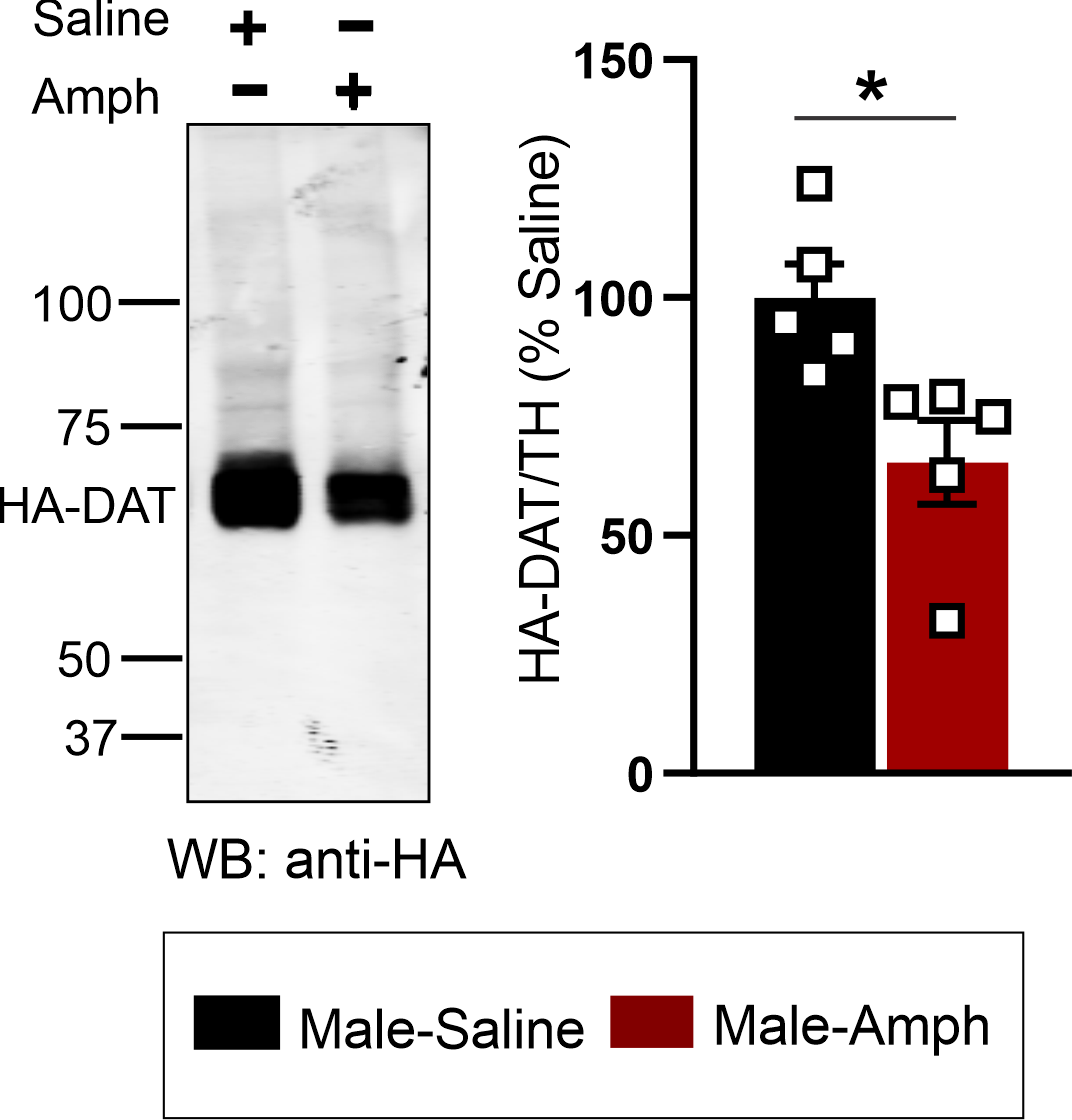

Supplement: Figure 2-1 — Amph challenge of Amph-sensitized mice decreases HA-DAT protein level in striatum of males. Striatal synaptosomes obtained after saline and Amph-challenged on Day 14 from male mice were lysed as in Figure 2A. The protein samples [60 µg/ lane of striatal synaptosomes] were resolved by 7.5% SDS-PAGE, transferred to nitrocellulose and probed with HA and TH antibodies by Western blotting. Representative blot is shown. The bar graph shows the mean (± S.E.M) intensity of bands from 3-4 independent experiments. The amounts of HA-DAT were normalized to the amounts of TH and expressed as percent of the normalized amount in Saline samples. Asterisks indicate significant differences compared to Saline group, *p ˂ 0.05 (Student’s unpaired t-test [n = 5]). Download Figure 2-1, TIF file. [file eneuro-11-ENEURO.0491-23.2023-s002.tif]

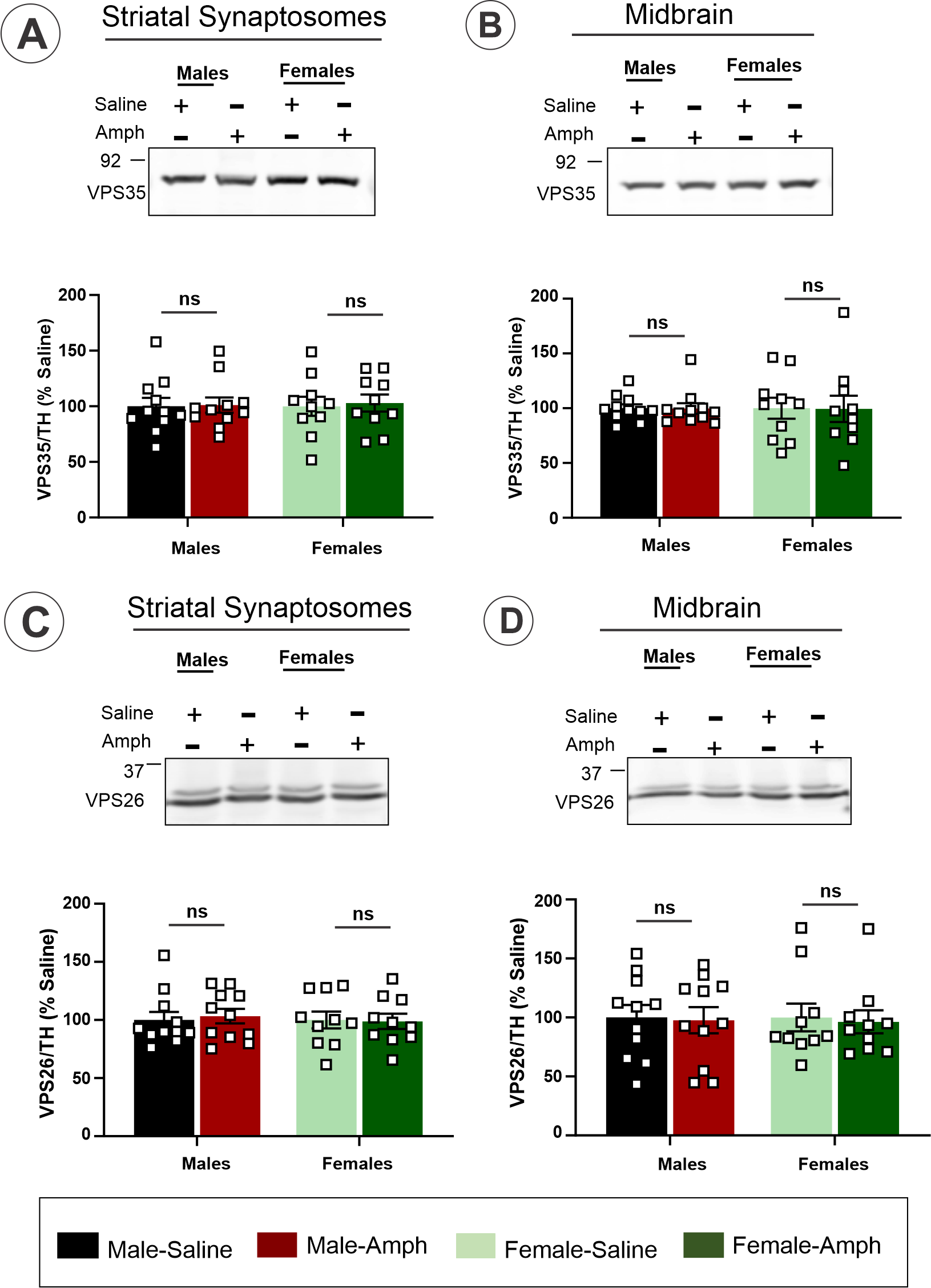

Supplement: Figure 4-1 — Amph challenge does not affect the amounts of retromer complex in sensitized mice. Striatal synaptosomes (A-B) and total MB tissue lysates (C-D) were electrophoresed and probed by immunoblotting with antibodies to VPS35 (A and C), VPS26 (B and D). Representative immunoblots are shown. Bar graphs represent mean values (with SEMs) of VPS35/26 band intensities normalized to TH shown in Figure 2. Quantification of band intensities revealed no significant differences between male and female mice. The data are from three or more independent experiments. ns, no significant difference. Download Figure 4-1, TIF file. [file eneuro-11-ENEURO.0491-23.2023-s003.tif]

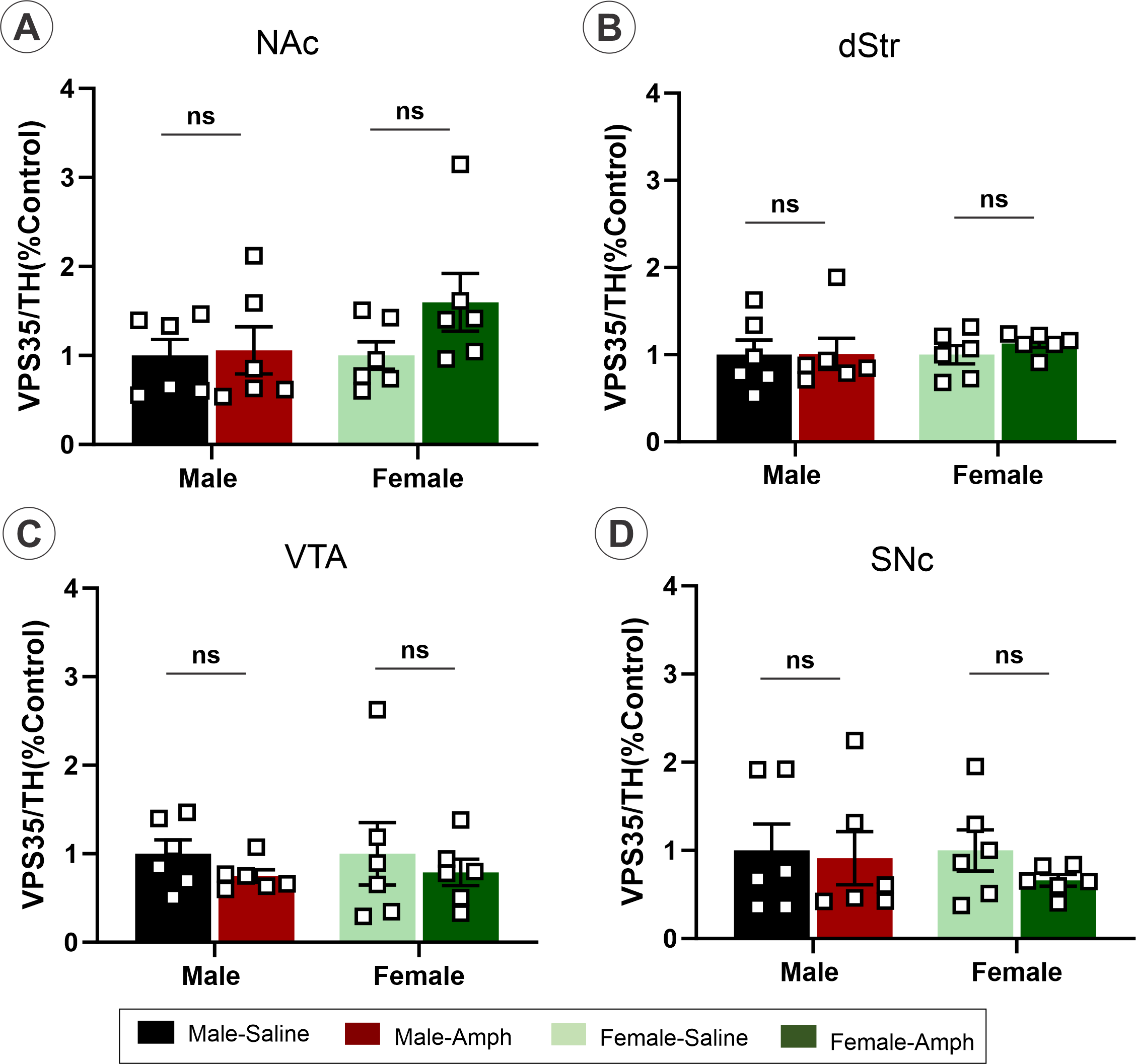

Supplement: Figure 7-1 — Amph challenge does not affect VPS35 levels in all brain regions tested in sensitized mice. Tissue lysates from NAc (A), dStr (B), VTA (C), and SNc (D) were electrophoresed and probed by immunoblotting with antibodies to VPS35 (A-D). Representative immunoblots are shown. Bar graphs represent mean values (with SEMs) of VPS35 band intensities normalized to TH. Quantification of band intensities revealed no significant differences between male and female mice. The data are from three or more independent experiments. ns, no significant difference. Download Figure 7-1, TIF file. [file eneuro-11-ENEURO.0491-23.2023-s004.tif]
